# Molecular Recognition and Scavenging of Arsenate from Aqueous Solution Using Dimetallic Receptors

**DOI:** 10.1002/chem.201404723

**Published:** 2014-10-22

**Authors:** Chris D Moffat, Dominik J Weiss, Arun Shivalingam, Andrew J P White, Pascal Salaün, Ramon Vilar

**Affiliations:** [a] Department of Chemistry, Imperial College London London SW7 2AZ (UK) E-mail: r.vilar@imperial.ac.uk; [b] Department of Earth Sciences and Engineering, Imperial College London London SW7 2AZ (UK); [c] Department of Earth, Ocean and Ecological Sciences, University of Liverpool Jane Herdman Building, Liverpool L69 3GP (UK)

**Keywords:** adsorption, heavy metals, arsenic, dimetallic receptors

## Abstract

A series of copper(II), nickel(II) and zinc(II) dimetallic complexes were prepared and their affinities towards arsenate investigated. Indicator displacement assays (IDAs) were carried out to establish the complexes with best affinities towards arsenate. A di-zinc complex (**3**) was selected and its arsenate-binding abilities investigated by isothermal titration calorimetry (ITC). The X-ray crystal structure of this metallo-receptor bound to arsenate is also reported, which allowed us to establish the binding mode between **3** and this oxyanion. Immobilising **3** onto HypoGel resin yielded a novel adsorbent (Zn–HypoGel) with high affinity for arsenate. Adsorption of arsenate from competitive solutions and natural groundwater was greater than that of the commercially used iron oxide Bayoxide E33. Zn–HypoGel could be efficiently and simply regenerated by washing with sodium acetate solution.

## Introduction

The identification of arsenic as a potent carcinogen in 1993 led the World Health Organisation (WHO) to revise the guideline for arsenic content of drinking water from 50 μg l^−1^ to 10 μg l^−1^.[1] Arsenic in drinking water is threatening the health of people in more than 20 countries around the globe[2] and it is estimated that over 200 million people are being unknowingly exposed to unsafe levels of arsenic in their drinking water.[3] The pH and redox state of the water body determine the species present; in natural waters the hydrogen arsenate anions H_2_AsO_4_^−^ and HAsO_4_^2−^ dominate.[4] Arsenic naturally occurs in shallow zones of groundwater in many countries; the worst cases being West Bengal, India and Bangladesh where concentrations of up to 5000 μg l^−1^ have been reported.[4] Additionally, anthropogenic pollution of surface and ground waters from mining and ore-processing effluents, insecticides, herbicides and wood preservatives is problematic.[4]

Existing methods for the removal of arsenates from water are based on precipitation, coagulation/flocculation, membrane filtration, and adsorption, with the latter being the most widely used.[2] However, most adsorbents suffer from fast saturation or poor selectivity and, therefore, many problematic waters with high levels of competing ions cannot be remediated through adsorption. Furthermore, there is a drive to improve upon the efficiency and arsenic affinity of adsorbents such as Bayoxide E33, which have already been successfully implemented at treatment plants for economic and environmental reasons, as well as health reasons. It has been suggested that drinking water containing arsenic at or below 10 μg l^−1^ still carries an increased risk of developing cancer.[5]

Polymeric ligand exchangers (PLEs) have been shown great promise in arsenic remediation.[6, 7] Though based on the principles of ion exchange, the active sites of some of these materials contain transition metal ions rather than quaternary ammonium groups as most traditional ion exchangers do. In principle, if the active site contains an oxyanion selective receptor, this should lead to a material with even greater arsenate affinity and selectivity.

There have been extensive studies of receptors (both organic and metal–organic)[8, 9] that act as phosphate binders, however, there are only a few such receptors that have been shown to interact with arsenate[10–13] and, consequently, the potential use of such receptors in arsenate remediation has been very sparingly explored.[12] Herein we report on the affinity of a series of dimetallic (with Cu^II^, Ni^II^ and Zn^II^) receptors for arsenate in aqueous solution. Considering that the interaction of a metallo-receptor with anions is dictated by a fine balance between the steric, electronic and electrostatic requirements of the metal complex, we decided to investigate complexes with zinc(II), nickel(II) and copper(II). These three metals have different Lewis acidity and therefore different anion affinity. Structural characterisation of two of these receptors bound to acetate and an arsenate derivative are presented. The best arsenate receptor (based on zinc(II)) was incorporated into a polymeric support to yield a material with improved properties for the removal of arsenate from contaminated water.

## Results and Discussion

### Synthesis and characterisation of metal complexes

**Figure 1:**
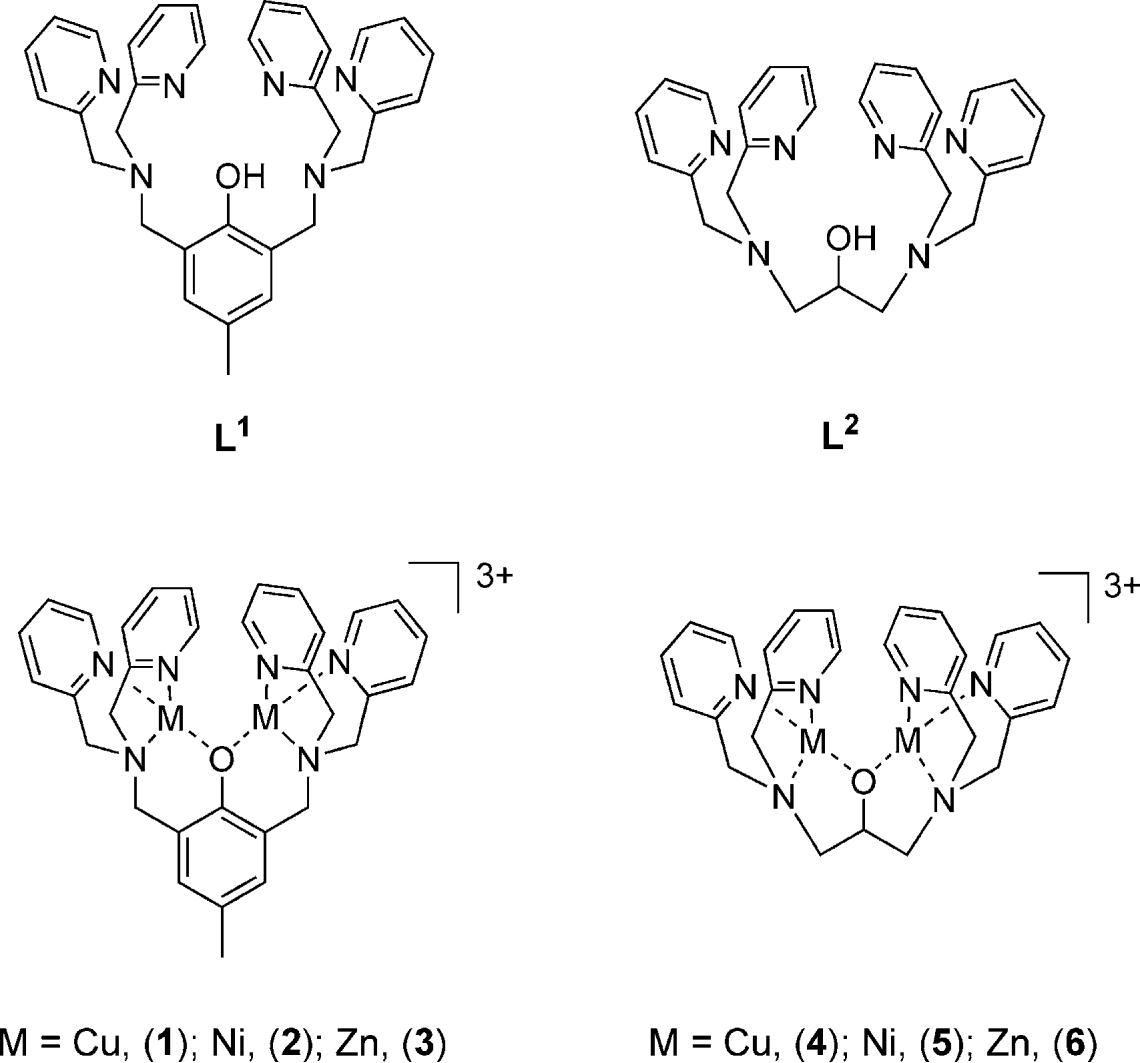
Chemical structure of L^1^ and L^2^ and the corresponding copper(II), nickel(II) and zinc(II) dinuclear complexes.

The syntheses of di-metallic complexes of ligands **L^1^** and **L^2^** (see Figure 1) have been reported before[14–17] and some of these complexes have been tested as metallo-receptors for oxoanions such as phosphate and acetate.[18] Therefore, we hypothesised that these types of complexes would be good candidates for the molecular recognition of arsenate. Thus, we synthesised both ligands, **L^1^** and **L^2^**, and the corresponding di-copper(II), di-nickel(II) and di-zinc(II) complexes (Figure 1). We initially prepared **L^1^** as reported,[19] but found this route troublesome and low-yielding. Therefore, we explored an alternative route based on the reaction between 2,6-bis(chloromethyl)-4-methyl phenol and di-(2-picolyl)-amine (DPA) in the presence of potassium iodide and a base. A key feature of this synthetic protocol is the omission of solvent; a high yield of the desired product was obtained, which could be used in subsequent metal-complexation reactions without the need for any further purification (see Experimental Section). Ligand **L^2^** was prepared in good yields using a previously reported method.[20]

The di-metallic complexes **1–4** and **6** were synthesised by slight modifications of previously reported procedures.[14, 16] However, the di-nickel(II) complex **5** had not been previously reported and was therefore prepared by adapting the synthetic protocol used for **4** and **6**. A hot (55 °C) methanolic solution of **L^2^** was first treated with a base (NEt_3_) and then mixed with Ni(OAc)_2_.The temperature was increased until reflux and NaPF_6_ was added to the reaction mixture. This was left to cool down to room temperature and then stored at −18 °C for two weeks. After this time, a small quantity of pink crystalline solid formed, which was removed by filtration to yield a dark blue solution. Diethyl ether was slowly diffused into this solution to yield the pure di-nickel(II) complex **5** as dark blue crystals. The compound was characterised by mass spectrometry, elemental analysis and X-ray crystallography.

### Structural determination of complex 5

**Figure 2:**
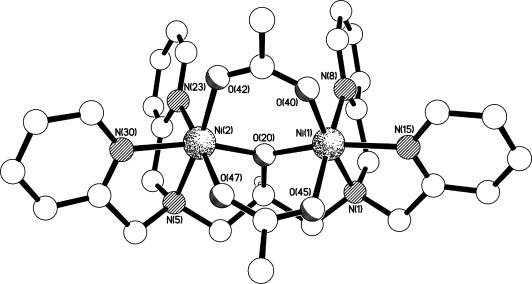
The structure of the di-nickel(II) cation present in the crystal of 5.

The crystallographic analysis confirmed the expected formulation for complex **5** (Figure 2). In this complex, **L^2^** binds to two nickel centres with approximate mirror symmetry about a plane that includes O(20) and bisects the Ni(1)⋅⋅⋅Ni(2) vector (Figure 2). The octahedral coordination spheres of each nickel atom are completed by two bridging acetates. The Ni–N (amine) bonds are slightly longer than their pyridyl counterparts [2.1108(12) and 2.1173(13) Å *cf*. 2.0625(13)–2.0913(13) Å], though this is made more complicated by a much greater variety amongst the latter (Table S1 in Supporting Information).

The included methanol solvent molecule is involved in an O–H⋅⋅⋅O hydrogen bond to the O(45) oxygen atom of one of the bridging acetate moieties, the O⋅⋅⋅O and H⋅⋅⋅O separations being 2.8213(17) and approximately 1.93 Å, respectively, with a O–H⋅⋅⋅O angle of approximately 171°. Though no structures of this ligand with nickel have previously been reported,[21] there are four structures of the 2-methyl pyridyl analogue binding to two nickel centres in a very similar fashion to that seen here.[22, 23] Three of these structures involve a bridging acetate (the fourth has a bridging phosphate), though none possesses the second bidentate bridge seen in **5**.

### Screening affinity of metallo-receptors by indicator displacement assays (IDAs)

The binding of arsenate, phosphate and sulfate in aqueous solution buffered with HEPES at pH 7.5 by compounds **1**–**6** was investigated by indicator displacement assays (IDAs). It has been reported that binding of pyrocatechol violet (PV) by the di-zinc complex **3** at physiological pH is accompanied by significant colour change, which is reversed upon addition of a guest anion such as phosphate.[18] The six metallo-receptors were each mixed with one equivalent of PV and the resulting mixtures used to assess the ability of **1–6** to interact with arsenate, phosphate and sulfate. The aim of these experiments was to assess quickly and semi-quantitatively whether any of the six metal complexes had potential as an arsenate receptor. Therefore, each of the receptors pre-bound to PV was treated with 1 and 10 equivalents of the corresponding anions. The displacement of PV was determined by monitoring the intensity of UV/Vis absorbance at 445 nm.

**Figure 3:**
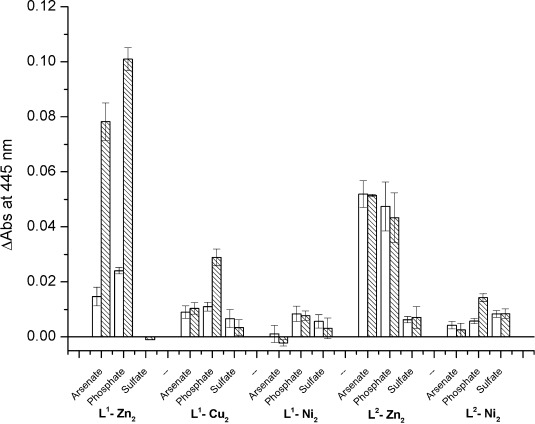
Plot showing the change in UV/Vis absorbance at 445 nm upon addition of 1 equivalent (white) and 10 equivalents (dashed) of each anion to a solution containing complex (50 μm) and PV (50 μm) in HEPES (100 mm) at pH 7.5 (note, no data is shown for L^2^–Cu_2_ as a precipitate formed upon mixing of this complex with PV).

As shown in Figure 3, the best receptor for arsenate and phosphate is the di-zinc(II) complex **3**, followed by **6**, also a di-zinc(II) compound. Interestingly, neither of these two metallo-receptors showed any binding to sulfate under the experimental conditions employed. Titrations of PV with each of **3** and **6** were used to determine the binding constant between PV and these complexes. Compound **3** showed an affinity for PV two orders of magnitude greater than **6** (see Supporting Information). Therefore, whereas complete displacement of PV from **6** was observed at lower anion concentrations than for **3**, this does not indicate that complex **6** binds the anions more strongly than complex **3** (see ITC data below for further discussion). On the other hand, addition of arsenate to the di-copper(II) and di-nickel(II) complexes pre-bound to PV did not lead to a significant displacement of the dye. Based on this data, the di-zinc(II) complexes **3** and **6** were chosen for further arsenate binding studies while the di-copper(II) and di-nickel(II) complexes were discarded. The affinity constants between these receptors and arsenate were then determined by IDA and isothermal titration calorimetry (ITC) (see next section).

### Interaction of 3 and 6 with oxoanions by isothermal titration calorimetry (ITC)

Isothermal titration calorimetry (ITC), an established technique to directly determine binding constants and yield thermodynamic information, was chosen to determine more accurately the interactions between metallo-receptors **3** (with two acetates and one BF_4_^−^ ion as counter-ions) and **6** (with one acetate and two PF_6_^−^ ions as counter-ions) with arsenate, phosphate and sulfate. Initial control experiments were carried out prior to determining the heat exchange associated to anion binding by the receptors. The corresponding anion was titrated into buffer (as well as buffer into buffer) and no significant signals were observed, which indicated no significant heat exchange upon addition of the anion salts to a cell containing only buffer. Therefore, in the presence of metallo-receptors, any heat exchange observed must be due to an interaction between the anion and the complex. Raw ITC data and the integrated binding curves for the titration of complex **3** with arsenate and phosphate can be found in the Supporting Information.

This data enabled the determination of the affinity-binding constants between **3** and these anions (Table 1). The titration data was fitted using a 1:1 model in MicroCal Origin software.[24] No data is reported for the interaction of **3** with sulfate because no binding was observed. These results are consistent with those obtained by IDAs, showing that the receptor binds with good affinity to arsenate and phosphate but not to sulfate.

**Table 1:** Apparent binding constants and enthalpy values determined for complex 3 by isothermal titration calorimetry (ITC), as well as binding constants determined by indicator displacement assay IDA (average of 3 titrations, errors shown are 1 standard deviation)

Anion	*K* [m^−1^] ITC	*K* [m^−1^] IDA	Δ*H* [kJ mol^−1^] ITC
Arsenate	(1.45±0.3) x 10^4^	(1.63±0.4) x 10^4^	−4.02±0.5
Phosphate	(2.08±0.5) x 10^4^	(2.10±0.4) x 10^4^	−3.68±0.2
Sulfate	No binding detected	No binding detected	n/a

The data presented in Figure 3 and Table 1 represents the interaction of the corresponding metallo-receptors with different protonation states of the anions. For each anion studied, the solutions were buffered at pH 7.5 using HEPES. At this pH, arsenate and phosphate are present as the mono- and di-protonated species: about 78 % HAsO_4_^2−^/22 % H_2_AsO_4_^−^ and about 66 % HPO_4_^2−^/34 % H_2_PO_4_^−^ (calculated using HyperQuad software[25] and the known p*K*_a_ values of these species). Therefore, the IDA data shown in Figure 3, as well as the affinity constants shown in Table 1 (determined by IDA and ITC), represent the interaction of the above mixtures rather than a single arsenate or phosphate species with the corresponding receptor. Because these equilibria in aqueous solution are well-established, it is rarely recognised when reporting affinity constants between oxoanions and chemical receptors in aqueous media. In the context of the studies presented, the apparent binding constants reported reflect more accurately the affinity of the complex for total arsenate in solution (such as in contaminated drinking water).

The ITC data was further used to determine Δ*H* for the binding process for both anions, which was negative, showing that these are enthalpy-driven interactions. A similar conclusion was reached by Han et al. on studying the interaction of complex **3** with phosphate.[18]

Under the ITC conditions described above, no significant interaction was detected between complex **6** and the anions presented here. This could be in part due to the poorer aqueous solubility of this complex; if the binding constants are less than those of complex **3**, then higher concentrations would be required for the heat exchange upon binding to be detected. However, the aqueous solubility of the complex was such that more concentrated solutions could not be prepared and, therefore, only anion binding by **3** could be quantified by ITC.

### X-ray crystal structure of 3 bound to an arsenate derivative

**Figure 4:**
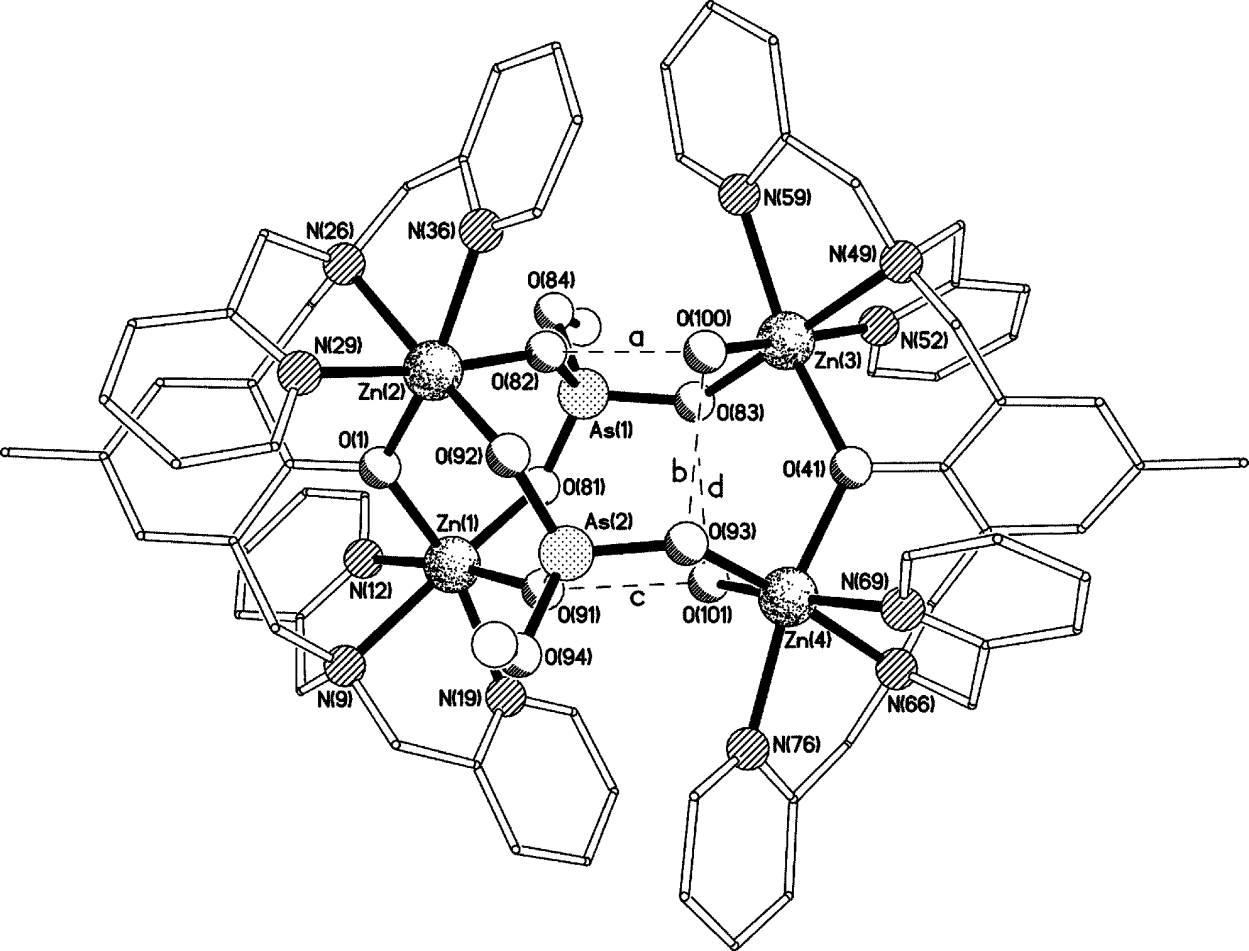
The structure of the di-cation present in the crystal of 7. The intramolecular hydrogen bonds a, b, c and d have O⋅⋅⋅O separations of 2.766(3), 2.767(4), 2.745(3) and 2.784(3) Å, respectively.

Having established that the di-zinc(II) complex **3** binds arsenate, it was of interest to obtain detailed molecular insight into the host–guest interaction. For this purpose, a methanolic solution of **3** was mixed with Na_2_HAsO_4_**⋅**7 H_2_O (2 equiv). Diethyl ether was then slowly diffused into the solution over one week at 4 °C. The structure of the resulting crystals showed that this product (**7**) comprises two [**L^1^**Zn_2_] units bridged by two AsO_3_(OMe) moieties (Figure 4). Interestingly, the bridges are asymmetric with both of the arsenate groups binding in a μ,κ^2^ fashion to the two zinc centres of one **L^1^**Zn_2_ unit, (the one containing Zn(1) and Zn(2), to the left of Figure 4) and simultaneously linking in a monodentate, κ^1^ manner to a metal centre of the other [**L^1^**Zn_2_] unit (the one containing Zn(3) and Zn(4), to the right of Figure 4). This gives the complex approximate *C*_2_ symmetry about an axis that passes through O(1) and O(41) rather than the *C_i_* symmetry that might have been expected. The “unused” coordination sites on Zn(3) and Zn(4) are occupied by water molecules [O(100) and O(101)], and these are involved in intramolecular hydrogen bonds to oxygen atoms of the arsenate groups (interactions **a**, **b**, **c** and **d** in Figure 4).

The As–O bond lengths (Table S2 in the Supporting Information) to the donor oxygen atoms show a pattern related to their coordination behaviour, with those to the oxygens involved in the μ,κ^2^ binding [O(81), O(82), O(91) and O(92)] being slightly shorter [in the range 1.659(2)–1.665(2) Å] than those to the oxygens involved in the monodentate coordination [O(83) and O(93), As–O distances 1.677(2) and 1.680(2) Å, respectively]. The methyl groups present in the two AsO_3_(OMe) moieties were presumably incorporated from the methanol that was used to grow the crystals. Previously, this zinc complex and similar transition metal complexes have been shown to catalyse esterification reactions of various phosphates.[26] Therefore, due to the similarity of arsenate and phosphate, it is plausible that a similar mechanism occurred here.

A search of the Cambridge Structural Database[21] for arsenate groups bridging two zinc atoms revealed no comparable structures; indeed, 16 of the 17 results comprised ZnAsO clusters of varying composition, the one exception incorporating tetradentate oxalate anions. Perhaps the closest previously determined structure is the zinc phosphate species bis((μ_3_-4-nitrophenylphosphato)-(μ_3_-2,6-bis(((2-methoxyethyl)(pyridin-2-ylmethyl)amino)methyl)-4-methylphenolato))-diaqua-tetra-zinc bis(hexafluorophosphate) (CCDC refcode VANTUB).[27] This has some variations to the septadentate ligand (two of the CH_2_–py arms changed to CH_2_CH_2_OMe units) and uses 4-nitrophenylphosphates instead of methoxyarsenates, but nevertheless shows a directly analogous dimerisation by the phosphates, even going so far as to have the same intramolecular hydrogen bonding pattern from the coordinated water molecules.

### Solid-supported di-zinc complex for arsenic scavenging

**Figure 5:**
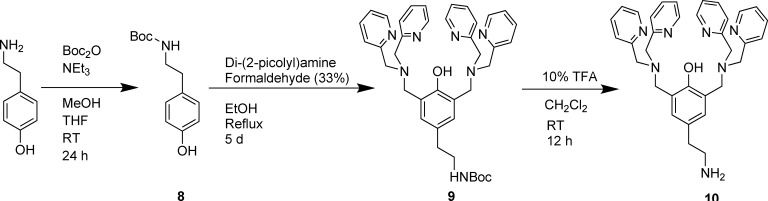
Synthesis of the amine-appended ligand to be immobilised on the resin. Procedure adapted from Kwon et al.[28] TFA=trifluoroacetic acid, Boc_2_O=di-*tert*-butyl dicarbonate.

After the solution and solid phase characterisation of the arsenate binding by complex **3** had been established, this metallo-receptor was immobilised onto a solid support with the aim of using the resulting functionalised material to remove arsenate from aqueous solution. HypoGel resin was chosen as solid support since the presence of PEG linkers in this resin gives it greater hydrophilic character than, for example, standard polystyrene resins. In addition, it contains carboxylic acid functionalities, which can be easily reacted with, for example, amines to attach a given molecule to the polymer beads. Thus, **L^1^** was functionalised with an ethyl amine (see Figure 5) following the procedure described by Kwon et al.[28] The resulting amine-functionalised **L^1^** (compound **10**) was then reacted with HypoGel resin containing 0.9 mmol g^−1^ loading of succinic acid.

**Figure 6:**
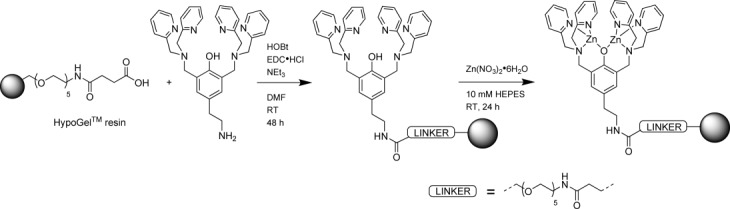
Synthesis of the functionalised resin using standard amide-coupling methods.

This reaction was carried out using standard amide coupling reagents in DMF, as shown in Figure 6. Following the reaction, the resin was washed with DMF, CH_2_Cl_2_, methanol and diethyl ether, and dried under reduced pressure until the weight remained constant. From the increase in dry weight of the resin, the extent of **L^1^** functionalisation was determined to be 0.25 mmol g^−1^. The functionalised HypoGel beads were then loaded with zinc(II) by shaking a suspension of beads in a solution of Zn(NO_3_)_2_**⋅**6 H_2_O (10 mm HEPES at pH 7). The zinc(II) concentration in the initial and final solutions was quantified using a colorimetric dye (pyrocatechol violet, see the Supporting Information). This resulted in a zinc(II) loading on the beads of 0.46 mmol g^−1^, that is, corresponding to 91 % of the ligand sites being filled with zinc(II).

### Batch equilibrium arsenate adsorption studies

Arsenate adsorption properties of the resin were studied by carrying out batch equilibrium adsorption experiments. The sorbent was shaken with a range of solutions of varying arsenate concentration (1 to 25 mg l^−1^) for 24 h, and arsenate concentrations left in solution were subsequently determined using electrochemical methods (a preliminary kinetic experiment confirmed that equilibrium was reached by this point, see the Supporting Information). The solid/solution ratio was constant at 1 mg mL^−1^ and the pH was kept at 7 by buffering with HEPES. The arsenic uptake was compared with that of an iron oxide material (named Bayoxide E33) which is used commercially as an arsenic adsorbent.

The isotherm data was fit in Origin using the Langmuir equation shown below [Eq. (1)], where *q*_e_ is the amount of arsenic adsorbed at equilibrium (mg g^−1^), *C*_e_ is the concentration of arsenic in solution at equilibrium (mg l^−1^), *Q*_max_ is the theoretical arsenic adsorption capacity and *b* is the affinity coefficient.[29]


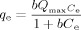
(1)

**Figure 7:**
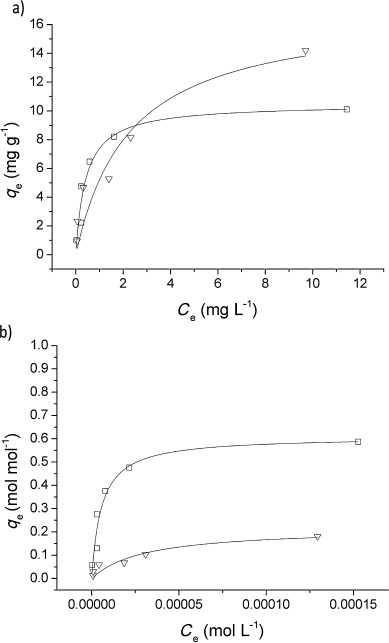
a) Arsenate adsorption isotherms with Langmuir fittings for Zn–HypoGel (□) and Bayoxide E33 (▿). In both cases, the adsorption was carried out using HEPES (10 mm) solutions (pH 7); b) arsenate adsorption isotherms for Zn–HypoGel and Bayoxide E33 shown in terms of the number of moles adsorbed per mole of active sites available. The latter was determined as previously reported by Kanematsu et al.[30]

Figure 7 a shows the Langmuir-fitted arsenate adsorption isotherms for the Zn–HypoGel resin and Bayoxide E33. From this data, the Zn–HypoGel *Q*_max_ was determined to be (10.2±0.6) mg g^−1^ and the value of *b* was (4.1±3.4) L mg^−1^ (average of 3 independent runs). Compared with other reported metal-based ligand-exchange type sorbents,[7] Zn–HypoGel has a moderate maximum capacity and a high arsenic affinity. For comparison, the arsenate adsorption isotherm for the commercial iron oxide is also shown in Figure 7 a and the *Q*_max_ was (17.1±3.5) mg g^−1^ (average of 3 independent runs). The two isotherms are compared more closely in Figure 7 b where the arsenic uptake is normalised to the number of moles of sorbent sites. Determination of the number of Bayoxide E33 active sites present was based on calculations previously carried out by Kanematsu et al.,[30] and the number of Zn–HypoGel sites was calculated from the results of the zinc loading experiments. Figure 7 b illustrates the significantly greater arsenate affinity of Zn–HypoGel sites compared with Bayoxide E33, that is, 1 mol of Zn–Hypogel sites would adsorb 0.6 mol of arsenate, whereas 1 mol of Bayoxide E33 sites would adsorb less than 0.2 mol of arsenate.

The difference in affinity is further shown by comparing the arsenate adsorption from the solutions at the lower end of the concentration range studied. As discussed elsewhere,[31] the maximum adsorption capacity is not always instructive when seeking to compare behaviours at low concentrations. For example, Zn–HypoGel reduced an initial arsenate concentration of 1000 to 2 μg l^−1^ after 24 h, whereas with Bayoxide E33 the final concentration was 60 μg l^−1^ (i.e., 99.9 % adsorption with Zn–HypoGel compared with 94.0 % with Bayoxide E33). This shows the superior arsenate-adsorbing properties of our new functional polymeric material.

### Effect of pH on arsenate adsorption

Because contaminated waters can have a range of different pH values, it was of importance to establish the working range of our new functionalised beads. Thus, the arsenate uptake over a range of pH values was investigated by batch studies. It has been previously demonstrated that iron oxide adsorbents show greatest arsenate adsorption at low pH, when the sorbent surface is positively charged.

**Figure 8:**
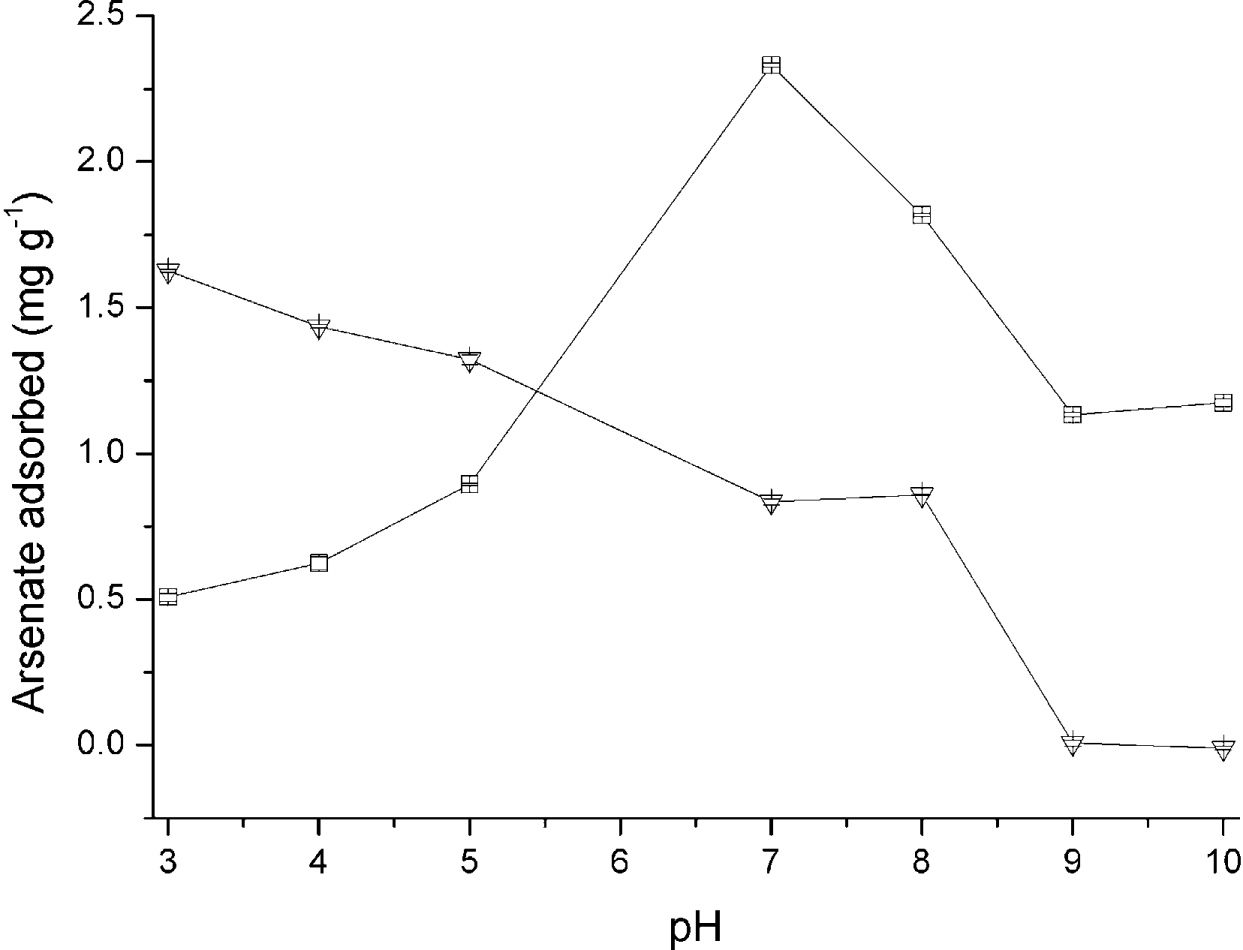
Plots showing the arsenic adsorbed on Bayoxide E33 (▿) and Zn–HypoGel (□) after shaking sorbent (5 mg) with arsenate solution (50 mL) for 24 h, with an initial arsenate concentration of 300 μg l^−1^.

The arsenate uptake over pH range 3 to 10 is shown in Figure 8. Bayoxide E33 performs best at low pH and adsorption is inhibited above pH 8.5 (the point of zero charge).[30] On the other hand, Zn–HypoGel resin demonstrates a greater affinity for arsenate above pH 5 and the adsorption is greatest at pH 7. This is advantageous as pH adjustment of natural water samples would not be required to reach the optimum performance of the Zn–HypoGel resin.

### Adsorption in the presence of competitive ions

**Figure 9:**
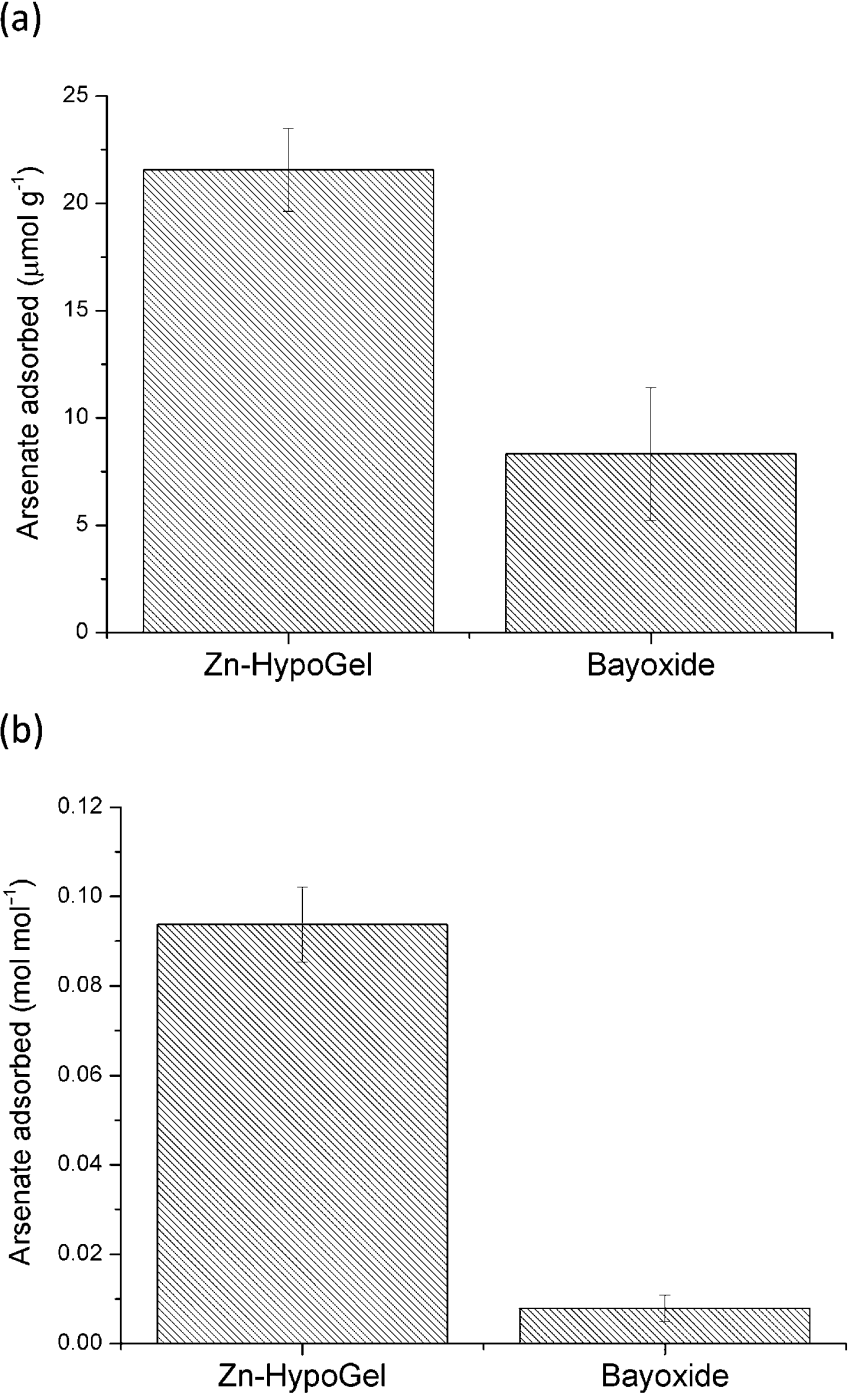
a) Number of moles of arsenate adsorbed onto Bayoxide E33 (5 mg) and Zn–HypoGel (5 mg) after 24 h of shaking in ′Challenge Water′ (50 mL) at pH 7; and b) number of moles of arsenate adsorbed per mole of active sites available.

To investigate the adsorption performance of the materials under more realistic conditions, batch experiments were carried out using solutions with a composition based on NSF Standard 53 (see Supporting Information for details of its composition),[32] so called “Challenge Water”, which is used as a standard in many laboratories to assess the performance of potential arsenic adsorbents. The resulting arsenate uptake from “Challenge Water” (at pH 7) after shaking both Zn–HypoGel and Bayoxide E33 for 24 h is shown in Figure 9 a. It can be seen clearly that 5 mg of the Zn–HypoGel sorbent was able to adsorb approximately three times more arsenic from this competitive solution than Bayoxide E33. This can be attributed to the greater selectivity of Zn–HypoGel adsorption sites towards arsenate over the majority of the species present; solution-phase studies of the zinc complex described above showed that only arsenate and phosphate would bind. Figure 9 b shows the same adsorption data in terms of the number of moles of sorbent active sites: Zn–HypoGel adsorbs approximately 18 times more arsenate per mole from the pH 7 “Challenge Water” than Bayoxide E33.

The arsenic uptake by both sorbents from “Challenge Water” adjusted to pH 5 was also carried out (see Figure S13 in the Supporting Information). As expected from the pH studies described above, Bayoxide E33 shows improved adsorption at reduced pH and adsorption by Zn–HypoGel is reduced. However, these studies also showed that even at this lower pH, Zn–HypoGel adsorbs more arsenate per mole of active sites.

### Adsorption from groundwater samples

**Figure 10:**
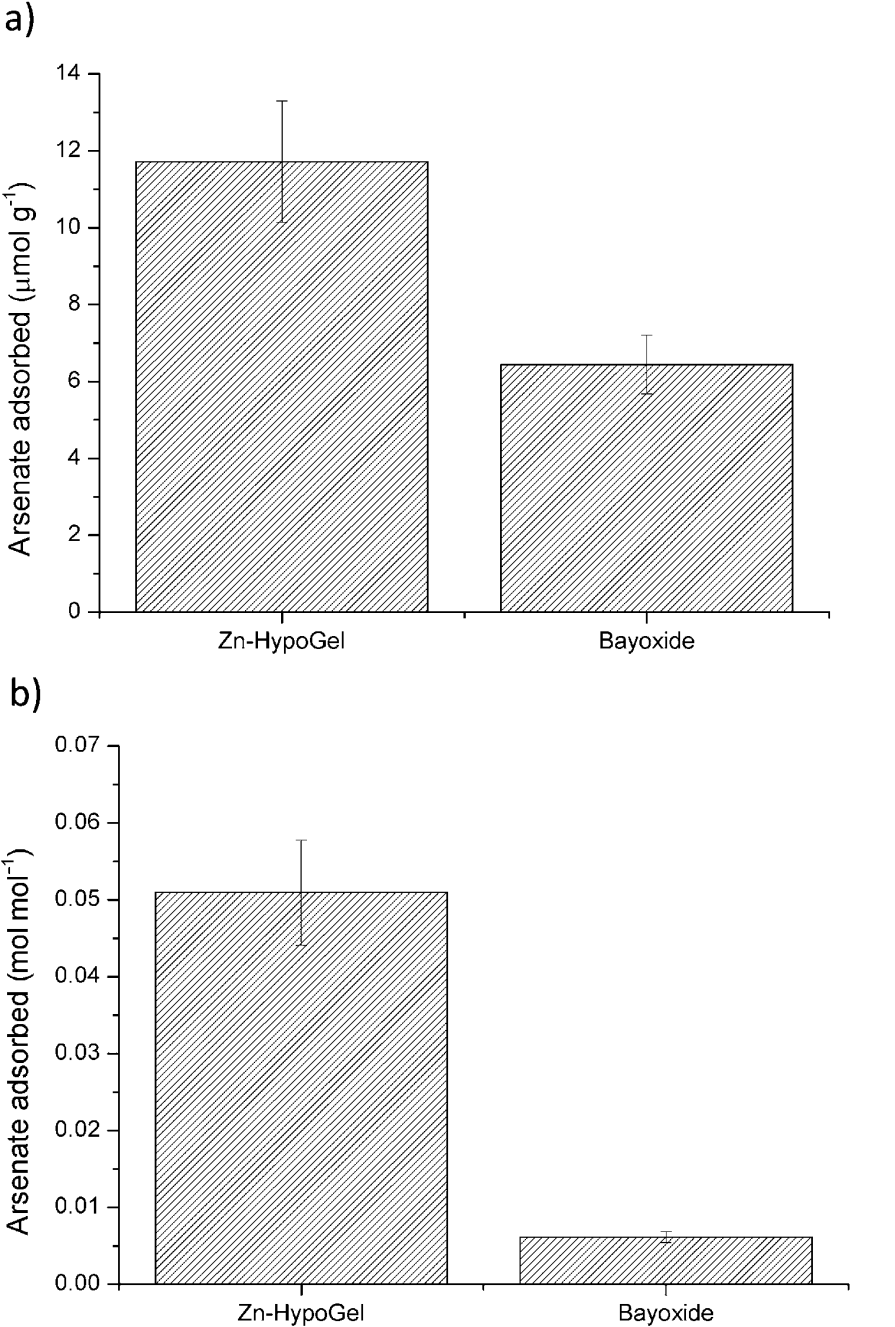
a) Number of moles of arsenate adsorbed onto Bayoxide E33 (5 mg) and Zn–HypoGel (5 mg) after 24 h of shaking in groundwater spiked with arsenate (1 ppm) at pH 7; and b) number of moles of arsenate adsorbed per mole of active sites available.

The viability of Zn–HypoGel for use in arsenic remediation was further investigated by testing the ability of this material to adsorb arsenate from natural groundwater samples. Water samples were taken from a water treatment plant in the UK, where arsenic remediation is routinely carried out. During the sampling process the water had been acidified to pH 1, therefore the pH was adjusted to 7.1 before the adsorption studies were carried out. The groundwater was spiked with 1 ppm arsenate and then shaken together with each of Zn–HypoGel and Bayoxide E33, with a solid-to-solution ratio of 1 mg mL^−1^. The arsenic uptake after 24 h is shown in Figure 10.

These experiments demonstrate that Zn–HypoGel could adsorb approximately two times more arsenate from a natural groundwater sample than Bayoxide E33.

### Regeneration of HypoGel resin

**Figure 11:**
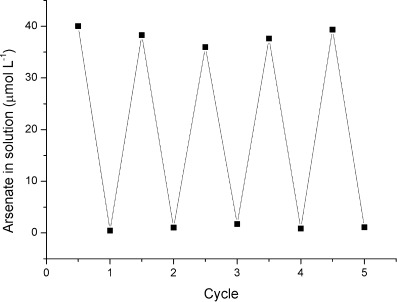
Plot showing adsorption and subsequent desorption (5 cycles) of arsenate from Zn–HypoGel resin. Resin (5 mg) was shaken with arsenic (5 mL of 40 μm, 3000 μg l^−1^) at pH 7. Desorption was carried out by shaking with sodium acetate solution (1 m, 5 mL) at pH 10.

To test the regeneration potential of the Zn–HypoGel sorbent, a series of adsorption/desorption cycles were carried out. Arsenate was first adsorbed at pH 7, as for the equilibrium studies. Desorption of the arsenate was successfully achieved by shaking with 1 m NaOAc, adjusted to pH 10, for 24 h. Basic brine solutions are well-known as regenerants for ion-exchange type sorbents, because the high concentration of chloride and hydroxide anions induces desorption of arsenate from the active sites.[6] Here, sodium acetate was used rather than chloride, because acetate forms a stronger interaction with the zinc complex than chloride, resulting in more efficient desorption of arsenate. However, the strength of acetate binding is still negligible compared with arsenate (see Figure S6 in the Supporting Information) and so subsequent re-adsorption of arsenate is not affected by the presence of bound acetate at the zinc centres. Figure 11 shows that even after multiple desorption cycles, the ability of the resin to bind arsenate was not impaired.

## Conclusion

Six dinuclear metal complexes with Cu^II^, Ni^II^ and Zn^II^ were synthesised and fully characterised. Their binding affinity for arsenate, phosphate and sulfate was investigated showing that the di-zinc(II) complexes (in particular **3**) are the best metallo-receptors for arsenate. This is consistent with the well-established affinity of zinc(II) for oxoanions such as phosphate. An X-ray crystal structure was obtained, which revealed the mode of binding to the metal centre, which, to the best of our knowledge, is the first X-ray crystal structure showing arsenate bound to a zinc(II) metallo-receptor. Immobilising complex **3** on a polystyrene resin resulted in a functional material (Zn–HypoGel) with a high affinity for arsenate. Zn–HypoGel could adsorb arsenate efficiently over a wide pH range, with the maximum adsorption occurring at pH 7. Adsorption from solutions containing high levels of competing ions and from natural groundwaters demonstrates that Zn–HypoGel performs much better than Bayoxide E33 and therefore has potential for use in water remediation. Furthermore, Zn–HypoGel could also be regenerated and re-used by simply washing with a basic sodium acetate solution and showed no loss of performance even after multiple desorption cycles. Put together, these results show that our new functionalised polymeric material performs better than the currently used adsorbent Bayoxide E33.

## Experimental Section

### General

Ligand **L^2^**, metal complexes **1**–**4** and **6**, and compounds **8**–**10** were prepared by slight modifications of previously reported procedures (see the Supporting Information for details).

### 2,6-Bis((bis(pyridin-2-ylmethyl)amino)methyl)-4-methylphenol (L^1^)

This Finkelstein activation was carried out under solvent-free conditions. Di-(2-picolyl)amine (1.1 mL, 6 mmol), potassium iodide (0.499 g, 3 mmol), potassium carbonate (1.001 g, 3 mmol) and PEG-400 (0.1 mL, 0.3 mmol) were ground together with a mortar and pestle for 5 mins to form a yellow paste. 2,6-Bis(chloromethyl)-4-methylphenol (0.620 g, 3 mmol) was added and the resulting green paste was ground vigorously for 45 min, yielding a sticky green solid. This material was taken up in CH_2_Cl_2_ and washed with water until the aqueous layer was no longer cloudy. The organic layer was then dried overnight with sodium sulfate and filtered. The solvent was removed under reduced pressure to give a green oil (crude yield 88 %); this was recrystallised from 1:1 hexane/ether to give the desired product, **L^1^**, as a yellowish solid. (0.848 g, 1.6 mmol, yield 54 %); m.p.=95–97 °C; ^1^H NMR (400 MHz, CDCl_3_): *δ*=10.79 (s, 1 H, –OH), 8.54 (d, 4 H, *J*=4.5 Hz, Py–H), 7.62 (td, 4 H, *J*=7.8 Hz, 1.8 Hz, Py–H), 7.52 (d, 4 H, *J*=7.8 Hz, Py–H), 7.14 (m, 4 H, Py–H), 7.01 (s, 2 H, ArH), 3.89 (s, 8 H, PyCH_2_), 3.80 (s, 4 H, ArCH_2_), 2.25 (s, 3 H, ArCH_3_); ^13^C NMR (100 MHz, CDCl_3_): *δ*=159.3, 148.9, 136.5, 129.8, 123.7, 123.0, 122.0, 77.4, 77.1, 76.8, 59.8, 54.8; MS (ESI^+^): *m*/*z*: calcd for C_33_H_34_N_6_O: 531.29 [*M*+H]^+^; found: 531.29; elemental analysis calcd (%) for. C_33_H_34_N_6_O⋅0.5H_2_O: C 73.44, H 6.54, N 15.57; found C 73.81, H 6.38, N 15.34.

### 1,3-Bis(bis(pyridin-2-ylmethyl)amino)propan-2-olbis-nickel(II)hexafluorophosphatediacetate (5)

1,3-Bis(bis(pyridin-2-ylmethyl)amino)propan-2-ol (0.036 g, 0.08 mmol) was stirred in methanol (5 mL) at 55 °C. Triethylamine (0.012 mL, 0.08 mmol) was added and the hot solution was stirred for 10 min. Nickel acetate hydrate (0.032 g, 0.16 mmol) was then added and the temperature was raised to 67 °C. Sodium hexafluorophosphate (0.027 g, 0.16 mmol) was then added and the mixture heated at reflux for 30 min. The solution was then allowed to cool to room temperature and placed in the freezer (−18 °C) for 2 weeks. After this time, pink crystals had formed, which were removed by filtration. The mother liquor was then decanted and concentrated under nitrogen. This methanolic solution was then placed in the fridge, and after slow diffusion of diethyl ether the di-nickel product **5** was obtained as large deep-blue crystals suitable for X-ray analysis. (0.020 g, 0.022 mmol, 25 %); MS (ESI^+^) *m*/*z*: calcd for C_31_H_35_F_6_N_6_Ni_2_O_5_P**⋅**MeOH: 659 [M–PF_6_–OAc–H]^+^; found: 659; elemental analysis. calcd (%) for C_31_H_35_F_6_N_6_Ni_2_O_5_P**⋅**MeOH: C 44.38, H 4.54, N 9.70; found C 44.30, H 4.48, N 9.60.

### Crystal data for 5

[C_31_H_35_N_6_Ni_2_O_5_](PF_6_)**⋅**MeOH, *M*=866.08, monoclinic, *P*2_1_/*c* (no. 14), *a*=10.52058(6), *b*=14.41429(10), *c*=24.21878(16) Å, *β*=96.3228(6)°, *V*=3650.36(4) Å^3^, *Z*=4, *ρ*_cald_=1.576 g cm^−3^, μ(Cu_Kα_)=2.427 mm^−1^, *T*=173 K, blue/pale blue dichroic blocky needles, Oxford Diffraction Xcalibur PX Ultra diffractometer; 7118 independent measured reflections (*R*_int_=0.0214), *F*^2^ refinement,[33] *R_1_*(obs)=0.0282, *wR_2_*(all)=0.0732, 6437 independent observed absorption-corrected reflections [|*F*_o_|>4σ(|*F*_o_|), 2*θ*_max_=145°], 486 parameters.

### Crystal data for 7

[C_68_H_76_As_2_N_12_O_12_Zn_4_](BF_4_)_2_⋅2meOH, *M*=1902.43, monoclinic, *P*2_1_/*c* (no. 14), *a*=19.3412(3), *b*=14.3874(2), *c*=28.4490(3) Å, *β*=94.4691(12)°, *V*=7892.42(18) Å^3^, *Z*=4, *ρ*_cald_=1.601 g cm^−3^, *μ*(Mo_Kα_)=2.121 mm^−1^, *T*=173 K, colourless tablets, Oxford Diffraction Xcalibur 3 diffractometer; 26853 independent measured reflections (*R*_int_=0.0405), *F*^2^ refinement,[33] *R_1_*(obs)=0.0579, *wR_2_*(all)=0.1416, 17000 independent observed absorption-corrected reflections [|*F*_o_|> 4σ(|*F*_o_|), 2*θ*_max_=66°], 1073 parameters.

CCDC-995883 http://www.ccdc.cam.ac.uk/cgi-bin/catreq.cgi(**5**) and CCDC-995884 http://www.ccdc.cam.ac.uk/cgi-bin/catreq.cgi(**7**) contain the supplementary crystallographic data for this paper. These data can be obtained free of charge from The Cambridge Crystallographic Data Centre via http://www.ccdc.cam.ac.uk/data_request/cif

### HypoGel resin–ligand loading

HypoGel resin (amine acylated with succinic acid, loading: 0.9 mmol g^−1^, 0.203 g, 0.18 mmol) was swelled in 8 mL DMF. After 1 hour the solvent was removed by filtration. Compound **11** (0.200 g, 0.36 mmol), EDCI**⋅**HCl (0.041 g, 0.216 mmol), HOBt (0.033 g, 0.216 mmol) and triethylamine (0.062 mL, 0.45 mmol) were dissolved in DMF (8 mL) and this solution was shaken with the swollen resin at room temperature for 48 h. Ligand uptake was monitored by HPLC analysis. After 48 h the reaction solution was removed by filtration. The resin was washed once with 2 mL DMF and then with excess DCM, DMF, MeOH and Et_2_O, before being dried under vacuum until the weight no longer changed (0.233 g, 0.25 mmol g^−1^ loading, 33 %).

### HypoGel resin–zinc loading

HypoGel resin loaded with **11** (loading: 0.25 mmol g^−1^, 0.223 g, 0.059 mmol **11**) was shaken in 10 mm HEPES buffer at pH 7 (5 mL) for 30 min to allow the resin to swell. After the buffer was removed by filtration, the resin was shaken in a HEPES-buffered solution containing Zn(NO_3_)_2_**⋅**6 H_2_O (0.073 g, 0.24 mmol) at room temperature for 24 h. After this time the solution was removed by filtration, and the resin was washed 3 times with 3 mL of buffer (30 min each). The resin was then swollen in buffer again, and this loading and washing cycle was repeated twice more. After the final buffer wash, the resin was rinsed with Milli-Q water, methanol and diethyl ether and finally dried under vacuum. The zinc concentration in each of the starting and final solutions, as well as in the 3 buffer washes, was quantified using UV/Vis spectroscopy in conjunction with a colorimetric zinc indicator (PV). These concentration values were used to determine the amount of zinc taken up by the resin. Zn content=0.46 mmol g^−1^ (91 % sites filled).

### Indicator displacement assays (IDAs)

5 mm stock solutions of each receptor were prepared in acetonitrile and stored at −18 °C. A solution of 100 mm 4-(2-hydroxyethyl)-1-piperazineethanesulfonic acid (HEPES) in Milli-Q water was adjusted to pH 7.5 with 2 m NaOH and used as buffer. A 5 mm stock solution of pyrocatechol violet (PV) indicator was prepared freshly as required using this buffer solution. Working solutions (500 μm) of receptors and indicator were prepared by a twenty-fold dilution in buffer. Working solutions (500 μm) of Na_2_HAsO_4_**⋅**7 H_2_O, Na_2_HPO_4_ and (NH_4_)HSO_4_ were also prepared in 100 mm HEPES at pH 7.5 by dilution of 50 mm stocks. The general procedure for carrying out indicator displacement assays was as follows. PV working solution (25 μL) and receptor working solution (25 μL) were added to a series of wells on a 96 well plate. Then, 1 and 10 equivalents of each anion were added to the corresponding wells, and each solution was made to a final volume of 250 μL with HEPES buffer. The absorbance of each solution was then recorded at *λ*=445 nm.

The general procedure for determining the binding constant for PV by a metal complex was as follows. Increasing amounts of **3** were added to 25 μm solutions of PV in HEPES buffer in a 96 well plate. A plot of receptor concentration versus absorbance at 445 nm yields a binding curve, which was fitted in Origin using a 1:1 binding model described by Hargrove et al.[34] Anion-binding constants were determined by preparing solutions containing a 1:1 ratio of **3** and PV (at 50 μm) in HEPES buffer, and then adding increasing amounts of either arsenate or phosphate working solution. A plot of anion concentration versus absorbance at 445 nm yielded a binding curve for each interaction. These data could be fitted in Origin using a displacement assay script reported by Hargrove et al.[34]

### Isothermal titration calorimetry (ITC)

Solutions of each receptor (0.2 mm) were prepared in HEPES buffer (100 mm) at pH 7.5, and stored at −18 °C. Solutions of Na_2_HAsO_4_**⋅**7H_2_O, Na_2_H_2_PO_4_ and (NH_4_)HSO_4_ (3 mm) were also prepared in 100 mm HEPES at pH 7.5 by dilution of 50 mm stocks. All solutions were filtered through a 0.45 μm syringe filter and degassed before use. The basic procedure for the ITC experiments was as follows: a solution of receptor was stirred at constant temperature in the calorimetric cell. A solution of anion was then accurately titrated into the cell, and the heat change upon each addition was measured (or rather, the power required to maintain constant temperature with respect to a reference cell was measured). This titration data was then integrated using MicroCal Origin software[24] to produce a binding isotherm, from which *K*, Δ*H* and the stoichiometric parameter *n* could all be determined. A control experiment was carried out where the anion was titrated into a cell containing no receptor, to ensure that interaction between the anion and the buffer did not involve a significant heat exchange.

The titration data was fit using the following 1:1 model shown in Equation (2).


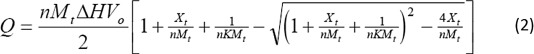
(2)

Where *Q*=heat content of the solution, *K*=the binding constant, *n*=number of sites, *V*_0_=active cell volume, *M_t_*=bulk concentration of host and *X_t_*=bulk concentration of ligand.

### Zinc analysis (UV/Vis analysis)

A working solution of Zn(NO_3_)_2_**⋅**6H_2_O (13.5 mm) was prepared in HEPES (10 mm) at pH 7. Aliquots of this zinc standard were then added to a cuvette containing PV (100 μm), and the absorbance spectrum recorded after each addition. This data was used to produce a calibration line of absorbance at 605 nm versus zinc concentration. Aliquots were then taken from the zinc loading solutions and mixed with PV (100 μm). The absorbance at 605 nm was recorded and the zinc concentration in the solutions could be determined.

### Arsenate adsorption isotherms

A solution of HEPES (10 mm) was prepared in Milli-Q water and adjusted to pH 7 with NaOH (1 m). An arsenic stock (1000 ppm) was prepared by dissolving Na_2_HAsO_4_**⋅**7H_2_O in 0.1 m HCl, and a 100 ppm solution was prepared by subsequent dilution of the stock. In a typical isotherm study, buffer (5 mL) was added to each of 7 Luer lock syringes, fitted with a frit and cap. Varying volumes of the arsenic stock and working solutions were then added to the syringes, to give an arsenic concentration range from 1 to 25 ppm. Adsorbent (5 mg) was then added to each syringe, and the solution placed on an orbital shaker at 100 rpm for 24 h. After this time, the shaking was stopped and each solution was removed and acidified with HCl (0.1 m). The arsenic concentration was then determined by differential pulse anodic stripping voltammetry (DPASV, see below).

### Regeneration of HypoGel sorbent

HEPES buffer (5 mL, pH 7) and arsenic stock (15 μL, 1000 ppm) were added to a Luer syringe fitted with a frit and lock, followed by HypoGel sorbent (5 mg). After 24 h of shaking at 100 rpm, the solution was removed by filtration and the concentration of arsenic remaining in solution was determined. A solution of NaOAc (1 m) was prepared and adjusted to pH 10 with NaOH (1 m). Acetate solution (5 mL) was added to the As-laden sorbent and the syringe returned to the shaker at 480 rpm. The arsenic concentration in the brine was determined after 24 h; this process was repeated until no further arsenic was desorbed. The resin was then washed with copious Milli-Q water and buffer, before the next arsenic solution was added, and the adsorption–desorption cycle was repeated.

### Electrochemical determination of arsenic

Total arsenic concentrations in the sample were determined by DPASV at a gold microwire electrode, similar to methods previously described by Salaün et al.,[5] but using differential pulse rather than square wave as the stripping technique. “Home-made” vibrating gold microwire (working electrode) and iridium wire (auxiliary electrode) electrodes were mounted onto a Metrohm VA stand. A Metrohm Ag/AgCl electrode was used as the reference. The diameter of the gold electrode was 25 μm, and it was cleaned twice daily by scanning the cyclic voltammogram 3 times in H_2_SO_4_ (0.5 m), using a deposition potential of −3 V for 30 s. In a typical ASV determination, the conditioning potential was 0.7 V for 3 s, the deposition potential was −1 V for 10 s. The ASV scan started at −0.2 V and ended at 0.1 V. The background current was determined either by scanning a cell containing only the background electrolyte (0.1 m HCl) or by scanning the sample using a deposition time of only 1 s. Two additions of a Fluka analytical standard were made to the sample and the concentration was determined using the standard addition method. Upon addition to the voltammetric cell, samples were acidified to pH 1 and, where necessary, diluted with Milli-Q water in order that the arsenic concentration was kept within the linear range during the standard addition procedure.

## Supporting Information

As a service to our authors and readers, this journal provides supporting information supplied by the authors. Such materials are peer reviewed and may be re-organized for online delivery, but are not copy-edited or typeset. Technical support issues arising from supporting information (other than missing files) should be addressed to the authors.

miscellaneous_information
