# Supplementary material for: Molecular Recognition and Scavenging of Arsenate from Aqueous Solution Using Dimetallic Receptors
Source: Chemistry. 2014 Oct 22;20(51):17168–77. doi: 10.1002/chem.201404723 (PMC4517099; doi:10.1002/chem.201404723)
Supplement: Supplementary file 1 — miscellaneous_information [file chem0020-17168-sd1.pdf]

CHEMISTRY
A European Journal

Supporting Information

© Copyright Wiley-VCH Verlag GmbH & Co. KGaA, 69451 Weinheim, 2014

Molecular Recognition and Scavenging of Arsenate from Aqueous Solution Using Dimetallic Receptors

Chris D. Moffat,[a, b] Dominik J. Weiss,[b] Arun Shivalingam,[a] Andrew J. P. White,[a] Pascal Salaün,[c] and Ramon Vilar*[a]

chem_201404723_sm_miscellaneous_information.pdf

Supporting Information

1. Indicator Displacement Assays

*Binding of PV by complex **3** – Job's Plot*

The method of continuous variance (Job's Plot) was used to confirm the stoichiometry of the receptor: interaction. A series of solutions containing **3** and PV in varying molar ratios (0:1, 1:9…9:1, 1:0) were prepared in a 96 well plate, and the UV/vis absorbance at 445 nm was read. As shown in Figure S1, a plot of mole fraction vs ΔAbsorbance yields a parabolic curve. According to the Job's method, the maximum (or minimum) of this plot occurs at the mole fraction that results in the maximum formation of the receptor: indicator complex. In this case, the minimum occurs at $\frac{[I]}{[R]+[I]} = 0.5$ so the ratio of receptor to indicator is 1:1 (or 2:2, 3:3 etc).

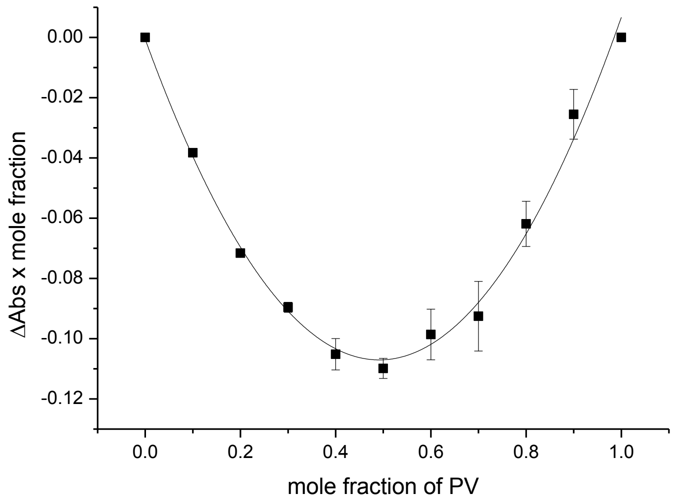

Figure S1, Job's plot constructed by measuring the UV/vis absorbance of a range of solutions containing varying molar ratios of **3** and PV. The minimum of the parabola occurs at 0.5

*Binding of PV by complex **3** – Titration*

The strength of the interaction between **3** and PV was investigated by UV/vis titration. Increasing amounts of **3** were added to 25 μM solutions of PV in a 96 well plate. A plot of receptor concentration vs absorbance at 445 nm yields a binding curve, which was fit in Origin using a 1:1 binding model described by Anslyn *et al* [1] and the binding constant was determined to be (2.3 ± 1) x 10^5 M^{-1}

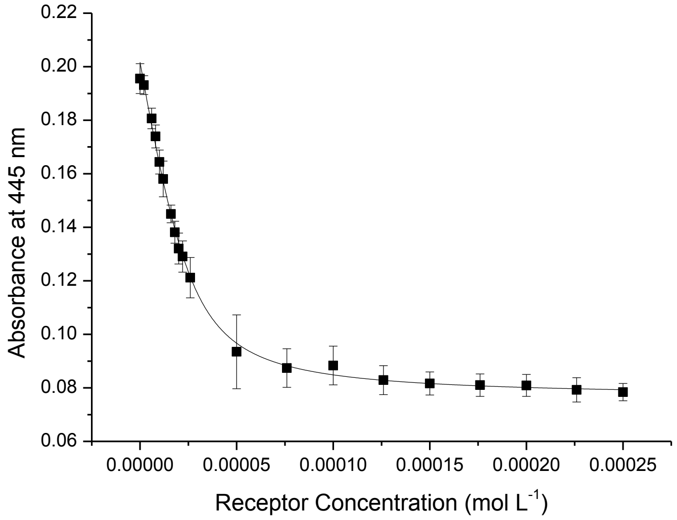

Figure S2, binding curve obtained upon titration of PV with complex **3** in 100 mM HEPES at pH 7.5, with fit curve shown in black

Binding of PV by complex ***6*** *– Titration*

The strength of the interaction between **6** and PV was investigated by UV/vis titration. Increasing amounts of **6** were added to 25 µM solutions of PV in a 96 well plate. A plot of receptor concentration vs absorbance at 445 nm yields a binding curve, which was fit in Origin using a 1:1 binding model described by Anslyn *et al* [1] and the binding constant was determined to be (7.5 ± 2.2) x 10^3 M^{-1}

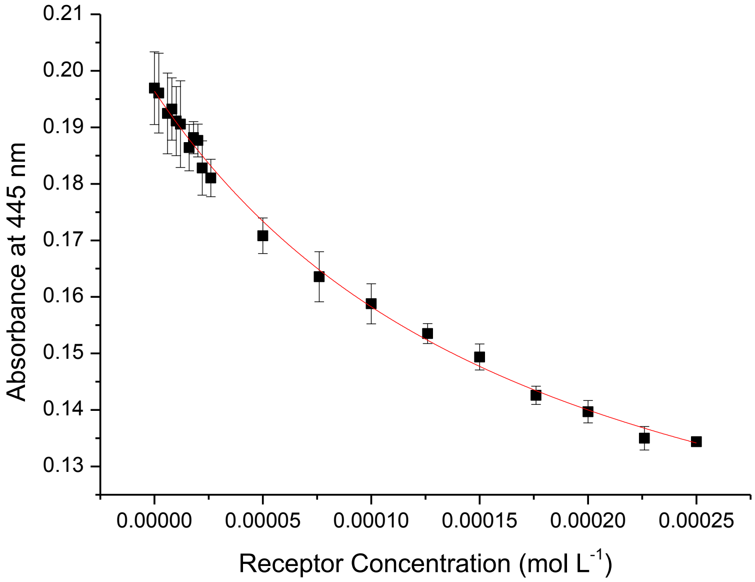

Figure S3, binding curve obtained upon titration of PV with complex **6** in 100 mM HEPES at pH 7.5, with fit curve shown in black

*Arsenate and Phosphate Binding by complex **3** – displacement of PV*

Displacement Assays could then be used to determine the strength of binding between **3** and arsenate and phosphate. Solutions containing a 1:1 ratio of **3** and PV were prepared in 100 mM HEPES at pH 7.5, and then increasing amounts of either arsenate or phosphate were added. Again, a plot of anion concentration vs absorbance at 445 nm yielded a binding curve for each interaction. These data could be fit in Origin using a displacement assay script reported by Anslyn *et al* and the anion binding constants were determined.

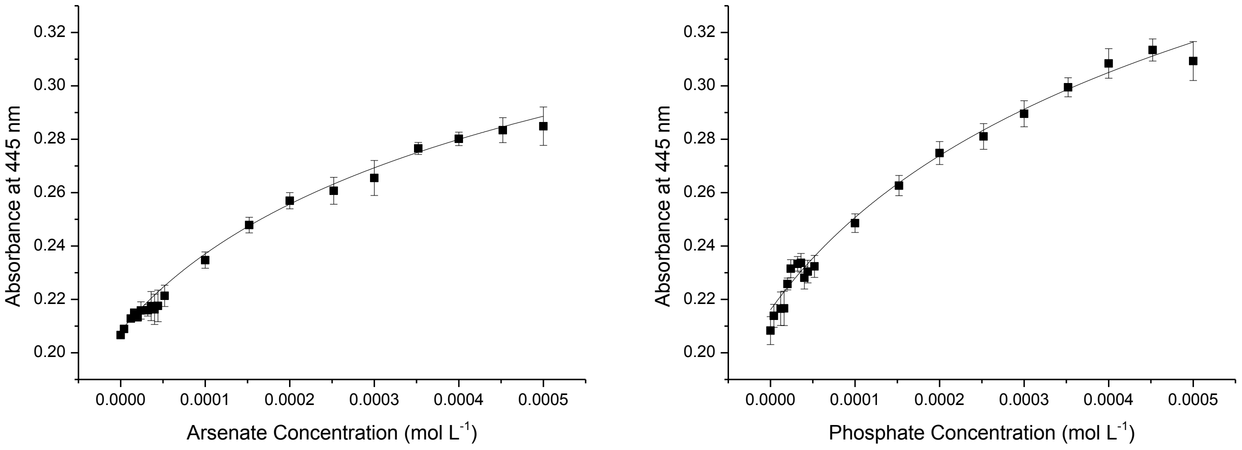

Figure S4, displacement curves obtained upon titrating a 1:1 mixture of **3** and PV with arsenate and phosphate in 100 mM HEPES at pH 7.5, with fit lines shown in black

The same method was also used to determine the strength of sulphate binding, however no displacement occurred even up to 10 equivalents of anion, and therefore the binding constant could not be determined.

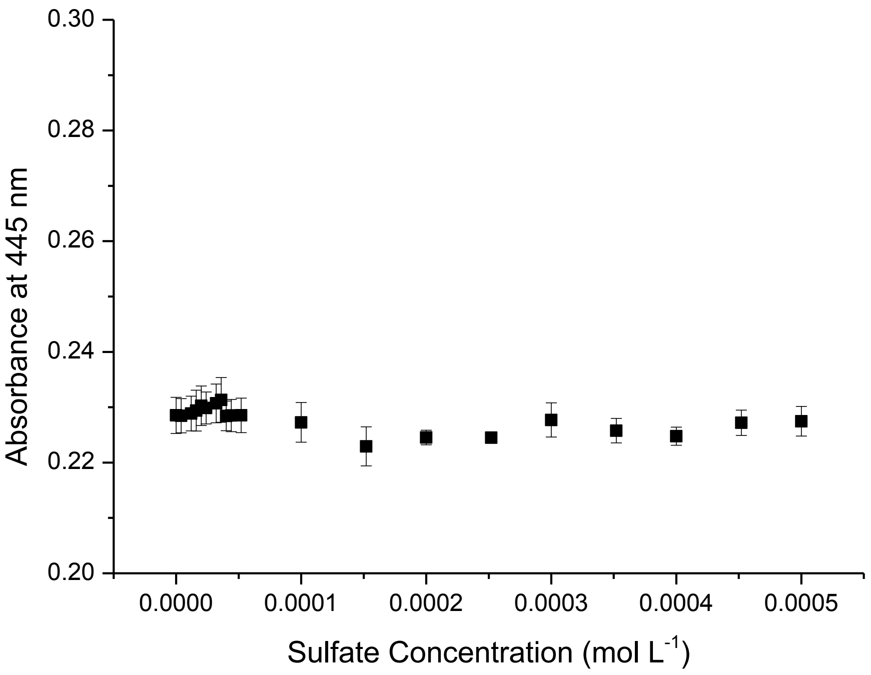

Figure S5, showing that no displacement occurred upon titrating a 1:1 mixture of **3** and PV with sulphate in 100 mM HEPES at pH 7.5

Displacement of PV by Acetate

As crystal structures of **3** have been reported with acetate counter-ion bridging the zinc(II) centres, and acetate (or other carboxylates) could be present in natural waters, displacement assays were also used to determine whether binding of acetate would be significant. Acetate was added to a solution containing a 1:1 mixture of **3** and PV.

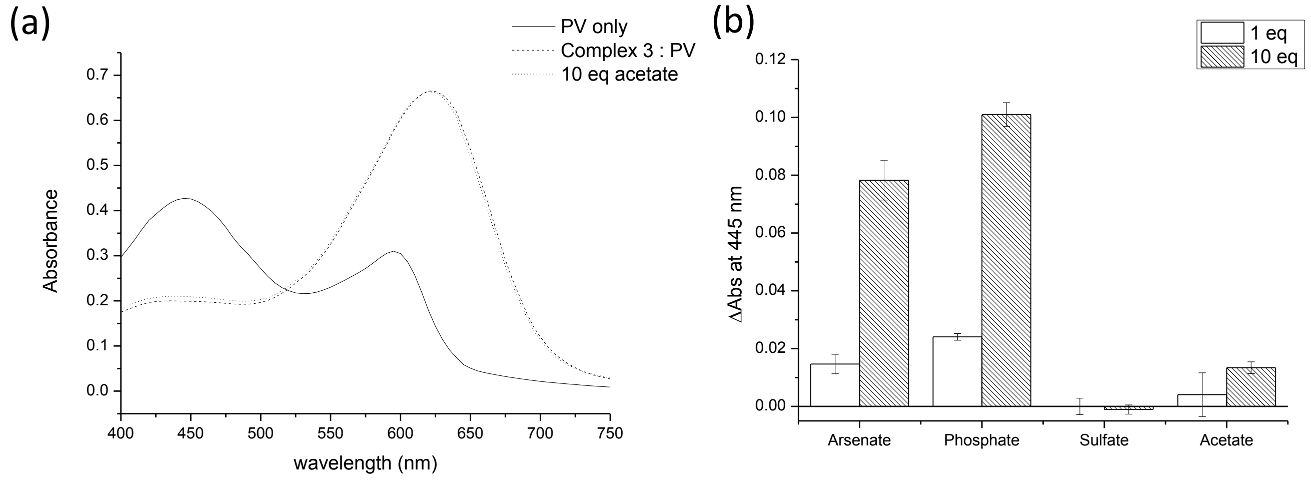

Figure S6, showing (a) UV-vis spectra of 50 µM PV, a 1:1 mix of **3** and PV (both 50 µM) and 50 µM **3**: PV in the presence of 10 equivalents of acetate; and (b) extent of displacement of PV by each anion. All solutions were prepared in 100 mM HEPES at pH 7.5

As shown in Figure S5, even addition of 10 equivalents of acetate to the solution induced only a tiny change in the UV-vis spectrum. This shows that binding of acetate is insignificant compared with arsenate or phosphate.

2. Isothermal Titration Calorimetry

*ITC – binding of Sulphate by complex **3***

ITC was also used to investigate sulphate binding by **3**, however as shown in figure S5, no binding interaction was observed.

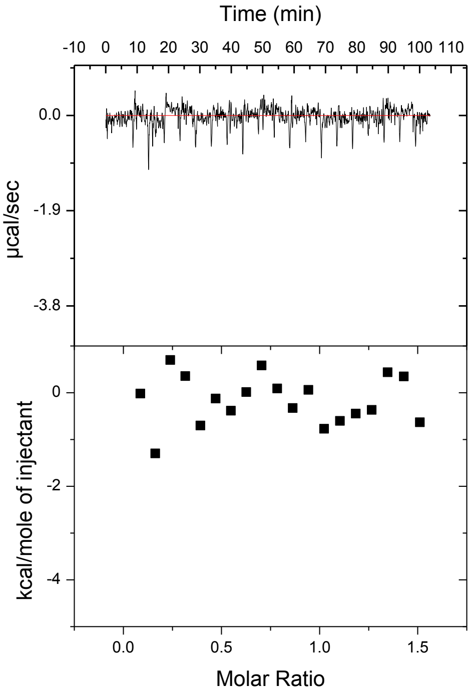

Figure S7, showing raw and integrated ITC data obtained upon titration of **3** with sulphate in 100 mM HEPES at pH 7.5

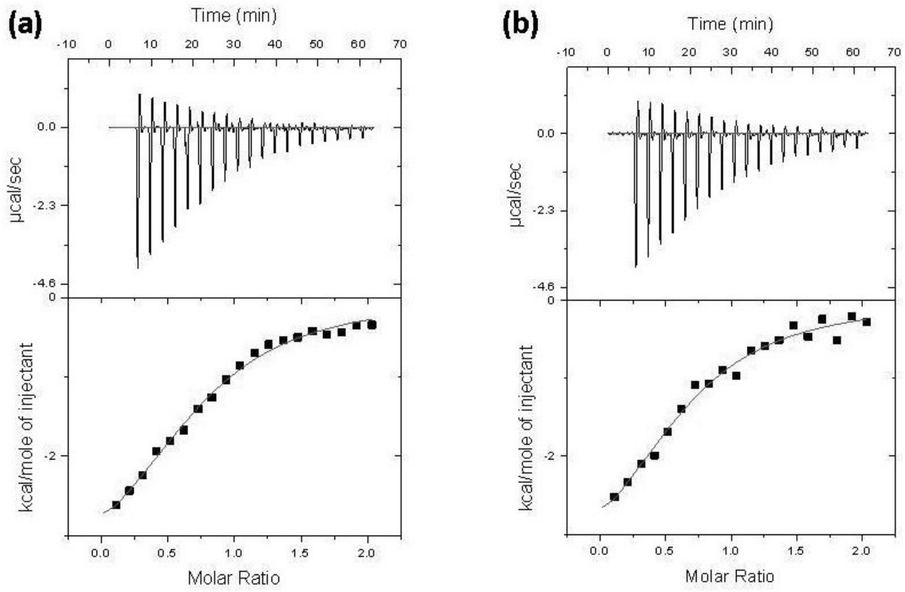

Figure S8 - ITC data and integrated binding curve obtained upon making: (a) 20 x 10 µL injections of 3 mM Na_2HPO_4 into a cell containing 0.2108 mM **3**. (b) 20 x 10 µL injections of 3 mM $Na_2HAsO_4 \cdot 7H_2O$ into a cell containing 0.2108 mM **3**. For both titrations, the spacing between each injection was 180 s and all solutions were prepared using 100 mM HEPES at pH 7.5.

3. Zinc Loading – Quantification

Zinc uptake by the functionalised HypoGel beads was determined by quantification of the zinc present in the initial and final reaction solutions, as well as the buffer washes. To this end, pyrocatechol violet was used as a zinc indicator. First, a zinc standard was used to produce a calibration curve for the response of PV to zinc in 10 mM HEPES at pH 7, as shown in figure S8.

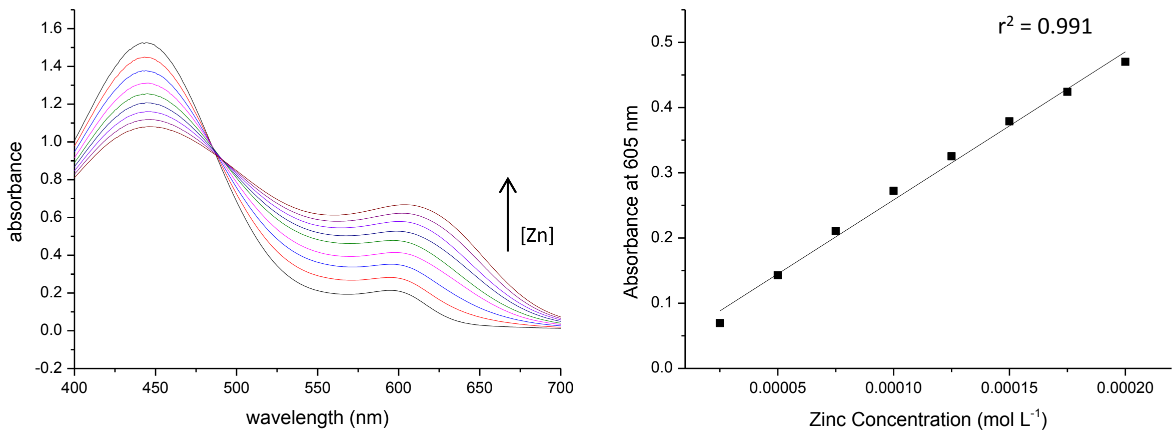

Figure S9, showing UV/vis spectra obtained upon titration of 100 μM PV with $Zn(NO_3)_2 \cdot 6H_2O$ in 10 mM HEPES at pH 7, and calibration line produced by plotting corrected absorbance at 605 nm vs zinc concentration

The zinc concentrations in the reaction solutions were then determined by mixing aliquots with 100 μM PV and recording the UV/vis absorbance. The absorbance at 605 nm could be directly related to zinc concentration by the calibration line shown in figure S8.

4. Adsorption – Kinetics Experiment

In order to confirm that arsenate equilibrium was reached during the batch adsorption studies, arsenate uptake by Zn-HypoGel was monitored over 24 hours. 5 mg of sorbent was added to a 50 ml solution containing 1800 ppb arsenate in 10 mM HEPES at pH 7. 100 μL aliquots were removed at each time point for subsequent voltammetric analysis.

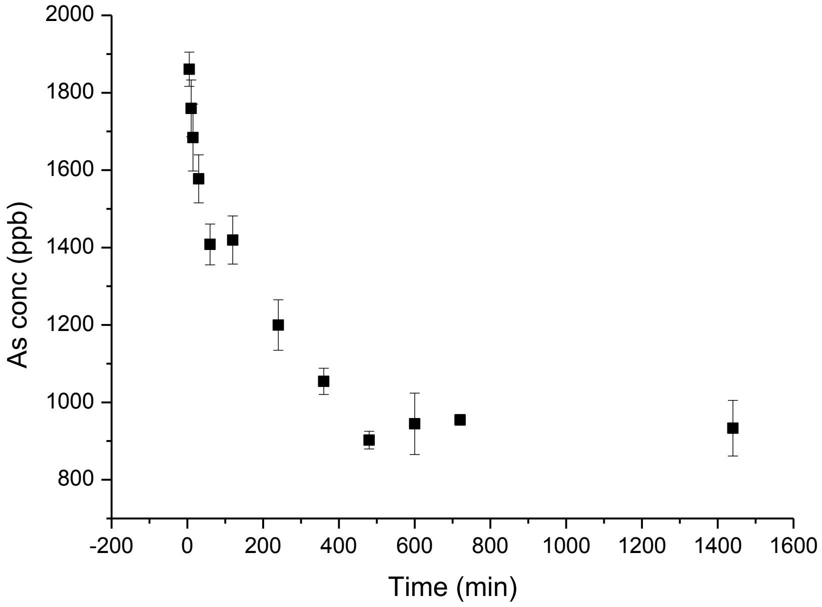

Figure S10, showing the change in arsenic concentration upon shaking 5 mg of Zn-HypoGel with 50 ml of 1800 ppb arsenate for 32 hours.

As can be seen in figure S9, there is no further change in concentration after 8 hours, and therefore equilibrium is reached well within the 24 time period of the batch experiments.

5. Crystallography

The X-ray crystal structure of ***5****.* The OH hydrogen atom of the included methanol solvent molecule in the structure of **5** was located from a ΔF map and refined freely subject to an O–H distance constraint of 0.90 Å. This group is involved in an O–H···O hydrogen bond to the O(45) oxygen atom of one of the bridging acetate moieties, the O···O and H···O separations being 2.8213(17) and ca. 1.93 Å respectively, with a O–H···O angle of ca. 171°.

The X-ray crystal structure of ***7****.* On the presumption that the two BF_4 anions located in the structure of **7** are the only counter ions present in this structure, the tetrazinc(II) complex must be a dication. (This presumption is complicated by the use of SQUEEZE (see below), but it was nevertheless clear that the difference electron density maps from before the use of SQUEEZE were inconsistent with the presence of any of the likely anions [BF_4, AsO_4 or OAc]). Given this, with each ligand being formally mononegative, then there must be four hydrogen atoms "missing" from somewhere amongst the two $MeOAsO_3$ units and the two

terminal oxygen atoms [O(100) and O(101)]. These hydrogen atoms could not be located from ΔF maps, but as both O(100) and O(101) appear to be involved in two intramolecular hydrogen bonds, the simplest and most likely explanation is that O(100) and O(101) are both H_2O ligands.

The included solvent was found to be highly disordered, and the best approach to handling this electron density was found to be the SQUEEZE routine of PLATON.[2] This suggested a total of 129 electrons per unit cell, equivalent to 32.3 electrons per molecule. The crystal was grown from a mixture of methanol [CH_4O, 18 electrons] and diethylether [$C_4H_{10}O$, 42 electrons], and before the use of SQUEEZE the electron density distribution most resembled methanol. 1.8MeOH equates to 32.4 electrons and this was rounded up to 2MeOH for simplicity and used as the solvent present. The atom list for the unit cell is thus low by 8(CH_4O), i.e. $C_8H_{32}O_8$. Combined with the presumed water hydrogen atoms on O(100) and O(101) not being located (4H low in the asymmetric unit, 16H low in the unit cell), the atom list for the unit cell is low by $C_8H_{48}O_8$ in total.

The O(94)-based methoxy group was found to be disordered. Two orientations were identified of ca. 75 and 25% occupancy, their geometries optimised, the thermal parameters of adjacent atoms restrained to be similar, and only the non-hydrogen atoms of the major occupancy orientation was refined anisotropically (the remainder were refined isotropically).

The B(1) and B(2)-based BF_4 anions were both found to be disordered. In each case three partial occupancy orientations were identified, (of ca. 53:25:22 and 59:24:17% occupancy respectively), their geometries optimised, the thermal parameters of adjacent atoms restrained to be similar, and only the atoms of the major occupancy orientations were refined anisotropically (the remainder were refined isotropically).

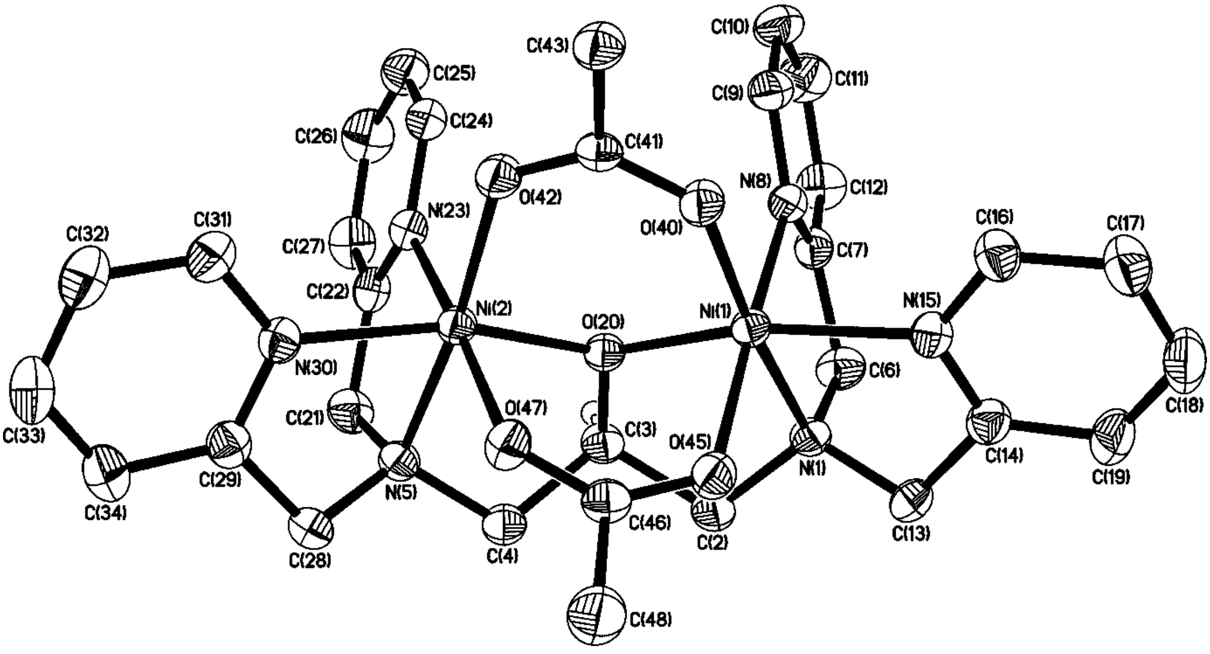

Fig. S11 The structure of the di-nickel(II) cation present in the crystal of **5** (50% probability ellipsoids)

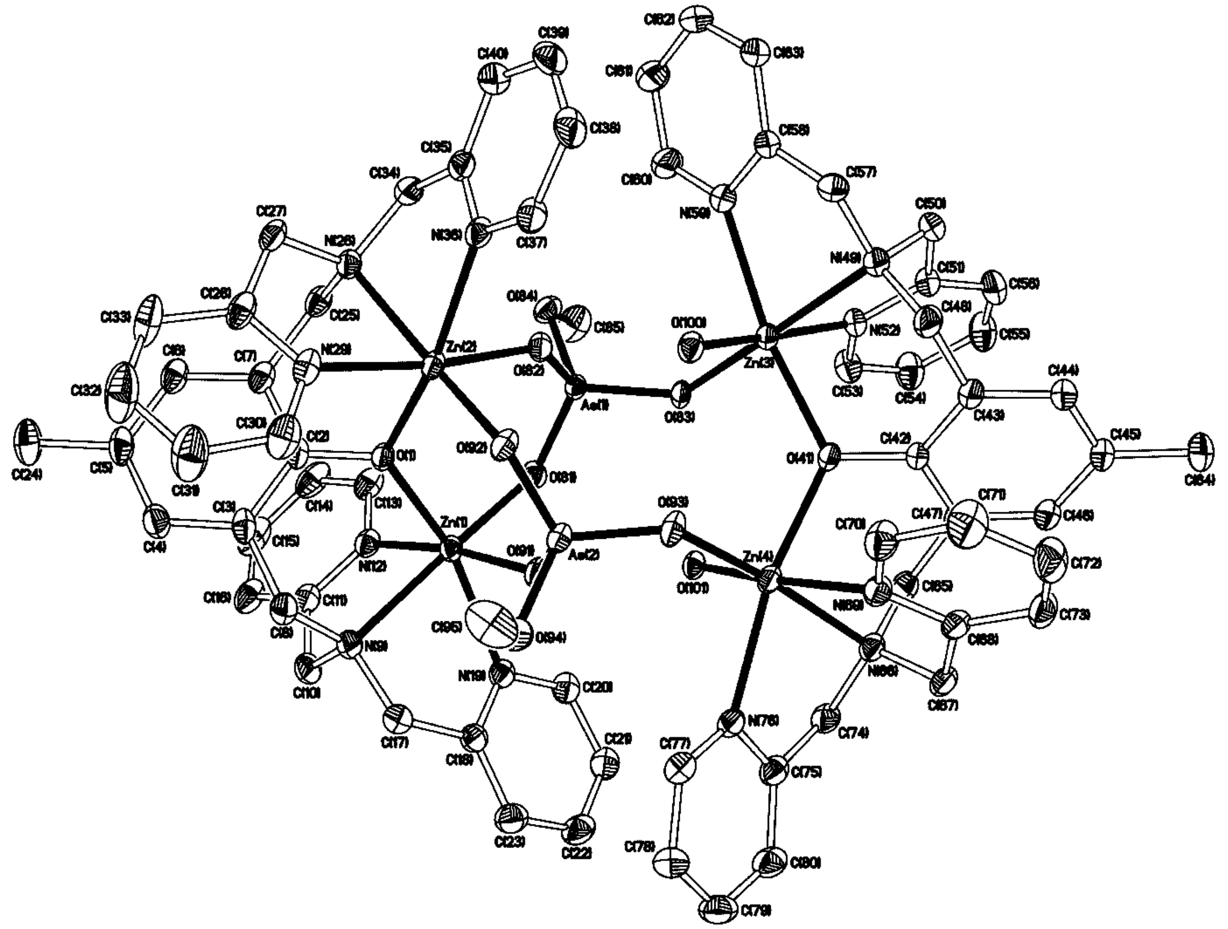

Fig. S12 The structure of the di-cation present in the crystal of **7** (50% probability ellipsoids)

Table S1 Selected bond lengths (Å) for the crystal structure of **5**.

Ni(1)–N(1)	2.1108(12)	Ni(2)–N(5)	2.1173(13)
Ni(1)–N(8)	2.0913(13)	Ni(2)–O(20)	1.9738(10)
Ni(1)–N(15)	2.0712(13)	Ni(2)–N(23)	2.0625(13)
Ni(1)–O(20)	1.9720(10)	Ni(2)–N(30)	2.0714(13)
Ni(1)–O(40)	2.0261(10)	Ni(2)–O(42)	2.0348(11)
Ni(1)–O(45)	2.1240(11)	Ni(2)–O(47)	2.0837(11)

Table S2 Selected bond lengths (Å) for the crystal structure of **7**.

Zn(1)–O(1)	2.053(2)	Zn(3)–O(83)	2.049(2)
Zn(1)–N(9)	2.229(3)	Zn(3)–O(100)	2.127(2)
Zn(1)–N(12)	2.177(3)	Zn(4)–O(41)	2.156(3)
Zn(1)–N(19)	2.228(3)	Zn(4)–N(66)	2.239(3)
Zn(1)–O(81)	2.019(2)	Zn(4)–N(69)	2.128(3)
Zn(1)–O(91)	2.096(2)	Zn(4)–N(76)	2.231(3)
Zn(2)–O(1)	2.064(2)	Zn(4)–O(93)	2.046(2)
Zn(2)–N(26)	2.257(3)	Zn(4)–O(101)	2.109(2)
Zn(2)–N(29)	2.176(3)	As(1)–O(81)	1.665(2)

Zn(2)–N(36)	2.198(3)	As(1)–O(82)	1.660(2)
Zn(2)–O(82)	2.076(2)	As(1)–O(83)	1.677(2)
Zn(2)–O(92)	2.036(2)	As(1)–O(84)	1.755(2)
Zn(3)–O(41)	2.144(2)	As(2)–O(91)	1.659(2)
Zn(3)–N(49)	2.230(3)	As(2)–O(92)	1.660(2)
Zn(3)–N(52)	2.132(3)	As(2)–O(93)	1.680(2)
Zn(3)–N(59)	2.238(3)	As(2)–O(94)	1.779(4)

6. Synthesis

2,6-bis((bis(pyridin-2-ylmethyl)amino)methyl)-4-methylphenol bis-copper(II) bistetrafluoroborate acetate (1). This complex was prepared by modification to a procedure described by Fenton *et al*[3]. $\mathbf{L^1}$ (0.159 g, 0.3 mmol) was stirred in MeOH (8 ml) at 55°C. NEt_3 (84 µL, 0.6 mmol) was then added to the solution, followed by $Cu(OAc)_2.H_2O$ (0.120 g, 0.6 mmol). The temperature was then raised to 65 °C and the solution stirred under reflux for 90 minutes. After this time, the stirring was stopped and the solution cooled to 45 °C. $NaBF_4$ (0.066 g, 0.6 mmol) was added. The solution was then shielded from light, slowly cooled to 0 °C and left to stand. After two days the solution was filtered and the solid was then washed with diethyl ether, giving complex **1** as a green solid. (0.151 g, 0.17 mmol, yield 57%); m.p. = 241 – 242 °C; MS (ESI^+) *m/z* 745.13 (calc for $[M\text{-}2BF_4\text{-}H]^+$ = 745.16, where M = $C_{33}H_{33}Cu_2N_6O$ $(OAc)(BF_4)_2.MeOH$); Elem. Anal. $C_{33}H_{33}Cu_2N_6O(OAc)(BF_4)_2.H_2O$ Calc. C 46.33, H 4.22, N 9.26; Found C 46.70, H 4.27, N 9.44%

2,6-bis((bis(pyridin-2-ylmethyl)amino)methyl)-4-methylphenol bis-nickel(II) tetrafluoroborate diacetate (2). This complex was prepared by modification to a procedure described by Fenton *et al*[3]. $\mathbf{L^1}$ (0.159 g, 0.3 mmol) was stirred in MeOH (8 ml) at 55 °C. NEt_3 (84 µL, 0.6 mmol) was then added to the solution, followed by $Ni(OAc)_2.4H_2O$ (0.149 g, 0.6 mmol). The temperature was then raised to 65 °C and the solution stirred under reflux for 90 minutes. After this time, the stirring was stopped and the solution cooled to 45°C. $NaBF_4$ (0.066 g, 0.6 mmol) was added. The solution was then shielded from light, slowly cooled to 0 °C and left to stand. After two days the solution was filtered to yield complex **2** as a blue solid, which was then washed with diethyl ether. (0.124 g, 0.14 mmol, yield 48%); m.p. = 333 - 335°C; MS (ESI^+) *m/z* 735.14 (calc for $[M\text{-}OAc\text{-}BF_4\text{-}H]^+$ = 735.17, where M = $C_{33}H_{33}Ni_2N_6O$ $(OAc)_2(BF_4).MeOH$) ; Elem. Anal. $C_{33}H_{33}Ni_2N_6O(OAc)_2(BF_4)$ Calc. C 52.16, H 4.61, N 9.86, Found C 51.80, H 4.52, N 9.69%

2,6-bis((bis(pyridin-2-ylmethyl)amino)methyl)-4-methylphenol bis-zinc(II) tetrafluoroborate diacetate (3). This complex was prepared by a modification to a procedure described by Fenton *et al.*[3] **L¹** (0.159 g, 0.3 mmol) was stirred in methanol (8 ml) at 55 °C. Triethylamine (0.084 ml, 0.6 mmol) was then added to the solution, followed by zinc acetate (0.110 g, 0.6 mmol). The temperature was then raised to 65 °C and the solution stirred under reflux for 90 minutes. The stirring was then stopped and the solution allowed to cool down to 45 °C. Sodium tetrafluoroborate (0.066 g, 0.6 mmol) was added, and the solution was shielded from light, slowly cooled to 0 °C and left to stand overnight. The resulting white precipitate was filtered from the solution and washed with diethyl ether and cold methanol, yielding **3** as a white solid. (0.137 g, 0.15 mmol, yield 51 %); m.p. = 309 – 310 °C; ^{1}H NMR (400 MHz, DMSO) δ 8.71 (d, *J* = 4.8 Hz, 2H, Py-*H*), 8.19 (d, *J* = 4.8 Hz, 2H, Py-*H*), 8.02 (td, *J* = 7.8, 1.6 Hz, 2H, Py-*H*), 7.62 (d, *J* = 7.8 Hz, 2H, Py-*H*), 7.53 (t, J= 7.8Hz, 2H, Py-*H*), 7.41 (td, *J* = 7.8, 1.6 Hz, 2H, Py-*H*), 7.13 (t, *J* = 7.8 Hz, 2H, Py-*H*), 6.55 (d, *J* = 7.8 Hz, 2H, Py-*H*), 6.44 (s, 2H, Ar*H*), 4.40 (d, *J* = 14.5 Hz, 2H, CH_2), 4.07 (d, *J* = 14.5 Hz, 2H, CH_2), 3.80 (d, *J* = 11.0 Hz, 2H, CH_2), 3.55 (d, *J* = 16.6 Hz, 2H, CH_2), 3.23 (d, *J* = 11.0 Hz, 2H, CH_2), 2.00 (s, 6H, O*Ac*), 1.95 (s, 3H, Ar-*Me*); MS (ESI$^+$) *m/z* 751.12 (calc for $[M\text{-}OAc\text{-}BF_4\text{-}H]^+$ = 751.16, where M = $C_{33}H_{33}Zn_2N_6O(OAc)_2(BF_4)$.MeOH); Elem. Anal. $C_{33}H_{33}Zn_2N_6O(OAc)_2(BF_4).2H_2O$ Calc. C 49.30, H 4.81, N 9.32; Found C 49.12, H 4.31, N 8.91

1,3-bis(bis(pyridin-2-ylmethyl)amino)propan-2-olbiscopper(II)bishexafluorophosphate acetate (4). This compound was prepared according to the method described by Fenton *et al.*[4] 1,3-bis(bis(pyridin-2-ylmethyl)amino)propan-2-ol (0.036 g, 0.08 mmol) was stirred in methanol (5 ml) at 55 °C. Triethylamine (0.012 ml, 0.08 mmol) was added and the hot solution stirred for 10 minutes. Copper acetate hydrate (0.032 g, 0.16 mmol) was then added and the temperature was raised to 67 °C. Sodium hexafluorophosphate (0.027 g, 0.16 mmol) was then added and the mixture heated at reflux for 30 minutes. The solution was then allowed to cool to room temperature and left to stand overnight. A light blue precipitate was formed – this was filtered off and washed with cold methanol and then pentane to yield the desired product **4** as a light blue solid. (0.037 g, 0.04 mmol, 50 %) MS (ESI$^+$) *m/z* 638 (Calc for $[M\text{-}2PF_6]^+$ = 638 where M = $C_{29}H_{32}Cu_2F_{12}N_6O_3P_2$); Elem. Anal. $C_{29}H_{32}Cu_2F_{12}N_6O_3P_2$ Calc. C 37.47, H 3.47, N 9.04, Found C 37.64, H 3.37, N 8.90.

1,3-bis(bis(pyridin-2-ylmethyl)amino)propan-2-ol bis-zinc(II) bishexafluorophosphate acetate (6). This compound was prepared according to the method described by Fenton *et al.*[4] **L^2** (0.05 g, 0.11 mmol) was stirred in methanol (8 ml) at 55°C. Triethylamine (0.015 ml, 0.11 mmol) was then added and the mixture stirred for 10 minutes. The temperature was increased to 67°C and zinc acetate (0.04 g, 0.22 mmol) was added. Sodium hexafluorophosphate (0.037g, 0.22mmol) was then added and the mixture heated at reflux for 30 minutes. The reaction was then allowed to cool to room temperature and left to stand overnight, yielding a white precipitate. This precipitate was filtered off and washed with cold methanol then diethyl ether, giving **6** as a white solid. (0.04 g, 0.04 mmol, 40 %); ^{1}H NMR (400 MHz, DMSO) δ 8.58 (dd, *J* = 12.2, 5.0 Hz, 4H, Py-*H*), 8.10 (m, 4H, Py-*H*), 7.67 – 7.52 (m, 8H, Py), 4.38 – 4.20 (m, 6H, NC*H*-Py), 4.03 (d, *J* = 16.9 Hz, 2H, NC*H*-Py), 3.74 (t, *J* = 11.0 Hz, 1H, C*H*(OH)), 3.06 (d, *J* = 12.06 Hz, 2H, NC*H*C), 2.04 (t, *J* = 11.5 Hz, 2H, NC*H*C); MS (ESI$^+$) *m/z* 673 (calc for $[M\text{-}2PF_6\text{-}H]^+$ = 673 where M = $C_{29}H_{32}F_{12}N_6O_3P_2Zn_2$.MeOH); Elem. Anal. $C_{29}H_{32}F_{12}N_6O_3P_2Zn_2$ Calc. C 37.32, H 3.46, N 9.00, Found C 37.14, H 3.31, N 8.77.

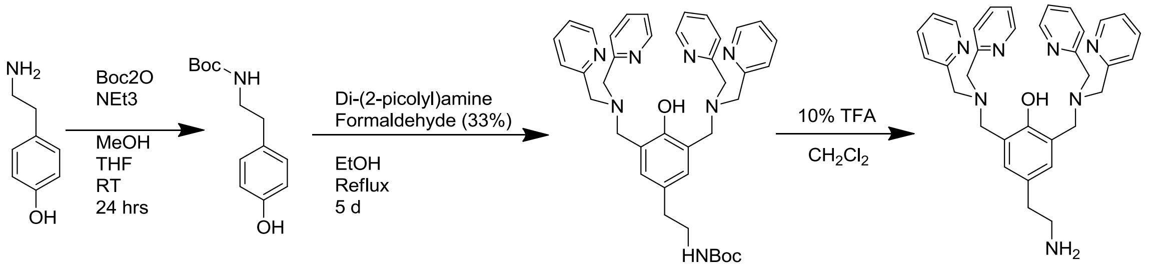

8 9 10

Boc-protected Tyramine (8). This compound was prepared according to the literature procedure described by Kwon *et al.*[5] Tyramine (1.003 g, 7.29 mmol) was stirred in THF (50 ml) and MeOH (10 ml) at 0 °C. NEt_3 (1.02ml, 7.29 mmol) was added and the mixture stirred at 0 °C. Boc_2O (1.585 g, 7.29 mmol) was dissolved in a small amount of MeOH and added dropwise to the stirring solution. The mixture was allowed to rise slowly to room temperature and then left to stir over night. The solvent was then removed under rotary evaporation and the resulting residue was taken up in ethyl acetate (~30 ml) and washed 3 times with 30 ml H_2O. The organic layer was then dried overnight with Na_2SO_4, filtered, and the solvent removed. The resulting brown oil was purified by silica column chromatography with 2:1 pentane/ ethyl acetate as the eluant to yield the desired product **7** as a white solid.

(1.485 g, 6.26 mmol, 86%); ^{1}H NMR (400 MHz, $CDCl_3$) δ 7.07 (d, *J* = 7.9 Hz, 2H, Ar*H*), 6.80 (m, 2H, Ar*H*), 5.16 (s, 1H, O*H*), 4.58 (s, 1H, N*H*), 3.36 (m, *J* = 6.6 Hz, 2H, CH_2), 2.74 (t, *J* = 7.2 Hz, 2H, CH_2), 1.46 (s, 9H, Boc); MS (EI$^+$) *m/z* 237 (Calc for [M^+] = 237.14)

Boc-protected 2,6-bis((bis(pyridin-2-ylmethyl)amino)methyl)-4-(2-aminoethyl)-phenol (9). This Mannich reaction was previously reported by Kwon *et al.*[5] A 36% formaldehyde solution (4.4 ml, 52.7 mmol) was stirred in ethanol (50 ml) at room temperature. Di-2-picolylamine (7.59 ml, 42.2 mmol) was dissolved in ethanol (10 ml) and added dropwise. The temperature was then raised and the mixture stirred at reflux (80 °C) overnight. Boc-protected tyramine (5 g, 21.1 mmol) was then added and the solution stirred at reflux for a further 5 days. The reaction was then allowed to cool to room temperature and the solvent removed under vacuum. The resulting residue was dissolved in ethyl acetate (60 ml) and washed 3 times with H_2O (60 ml). The organic layer was dried over Na_2SO_4, then filtered and the solvent removed. The resulting crude mixture was purified on a silica column with DCM/EtOH/NEt_3 (100:2:0.1) as eluent and finally recrystallized from 2:1 Pentane/Ethyl Acetate to yield the desired product **8** as a white solid. (4.700 g, 7.12 mmol, 34%); ^{1}H NMR (400 MHz, $CDCl_3$) δ = 10.94 (s, 1H, O*H*), 8.52 (d, *J*=4.8, 4H, Py-*H*6), 7.60 (td, *J*=7.72, 1.29, 4H, Py-*H*4), 7.48 (d, *J*=7.5, 4H, Py-*H* 3), 7.13 (t, *J* = 6.13, 4H, Py-*H* 5), 7.01 (s, 2H, Ar*H*), 3.88 (s, 8H, N-CH_2), 3.79 (s, 4H, Ar-CH_2-N), 3.34 (m, *J*=7.4, 6.8, 2H, CH_2), 2.70 (t, *J*=7.1, 2H, CH_2), 1.89 (s, 1H, N*H*), 1.40 (s, 9H, Boc); MS (EI$^+$) *m/z* 659 (Calc for $[M]^+$ = 659.36)

2,6-bis((bis(pyridin-2-ylmethyl)amino)methyl)-4-(2-aminoethyl)-phenol (10). The de-protection shown above was carried out according to a procedure described by Kwon *et al.*[5] Boc-protected 2,6-bis((bis(pyridin-2-ylmethyl)amino)methyl)-4-(2-aminoethyl)-phenol (1.172 g, 1.67 mmol)was stirred in dichloromethane (40 ml). As trifluoroacetic acid (4 ml, *excess*) was slowly added, the stirring solution became cloudy. After stirring overnight, the solvent was removed under vacuum. The resulting residue was then taken up in DCM (40 ml) and neutralised with saturated $NaHCO_3$ (aq) solution (40 ml) and then washed twice with H_2O (80 ml). The organic layer was dried over Na_2SO_4, filtered, and the solvent removed, giving the desired product **11** as a sticky brown solid. (0.815 g, 1.46 mmol, 82%); ^{1}H NMR (400 MHz, $CDCl_3$) δ = 10.92 (s, 1H, O*H*), 8.48 (d, *J*=4.9, 4H, Py-*H*6), 7.56 (td, *J*=7.6, 1.9, 4H, Py-*H*4), 7.42 (d, *J*=7.78, 4H, Py-*H*3), 7.09 (m, 4H, Py-*H* 5), 6.95 (s, 2H, Ar*H*), 3.82 (s, 8H, N-CH_2), 3.70 (s, 4H, Ar-CH_2-N), 2.99 (t, *J*=7.22, 2H, CH_2), 2.74 (t, *J*=7.21, 2H, CH_2); MS (ESI$^+$) *m/z* 560 (Calc for $[M+H]^+$ = 560.31)

7. Adsorption experiments

pH Adsorption Experiments. Solutions over a pH range of 3 – 10 were prepared by buffering Milli-Q water with 10 mM sodium formate (pH 3), sodium acetate (pH 4 and 5), HEPES (pH 7 and 8) and sodium tetraborate (pH 9 and 10). A 1000 ppm arsenic stock was prepared by dissolving $Na_2HAsO_4 \cdot 7H_2O$ in Milli-Q water. 50 ml of each buffer was added to separate reaction bottles, followed by 15 µL of As stock to give an initial arsenic concentration of 300 ppb. 5 mg of adsorbent was then added to the reaction solutions, and the bottles were placed on an orbital shaker at 100 rpm for 24 hours. After this time, the shaking was stopped and a sample removed and acidified with 0.1 M HCl, ready for analysis by DPASV.

Groundwater Adsorption Experiments. A groundwater sample was collected from a water treatment plant in the West Midlands and preserved by adjustment to pH 1 with HCl. The solution was then re-adjusted to pH 7 with 0.1 M NaOH and spiked with 1 ppm arsenate stock. 5 ml of this solution was then added to a luer syringe fitted with a frit and cap, followed by 5 mg of either adsorbent. The solution and sorbent were then shaken together on an orbital shaker at 100 rpm for 24 hours, after which time the solution was removed by filtration and acidified with 0.1 M HCl, ready for analysis by DPASV.

"Challenge Water" Adsorption Experiments. A solution consisting of Na_2SiO_3, $NaHCO_3$, $MgSO_4$, $NaNO_3$, NaF, $NaH_2PO_4 \cdot H_2O$, $CaCl_2$ and $Na_2HAsO_4 \cdot 7H_2O$ was prepared according to the procedure described for NSF Standard 53. The solution was adjusted to either pH 5 or pH 7 with 1 M HCl. In a typical experiment, 50 ml of this solution was added to a plastic bottle, followed by 5 mg of adsorbent. The bottle was then placed on an orbital shaker at 100 rpm for 24 hours, after which time the shaking was stopped and a sample removed and acidified

with 0.1M HCl. The arsenic concentration was then determined by Differential Pulse Anodic Stripping Voltammetry.

Table S3 - Concentration of each component of the NSF Standard 53 'challenge' solution, as well as their relative proportion to arsenate, which was present at 4.00 µM (300 µg L^{-1}).

Ion	Concentration (mM)	Equivalents (relative to arsenate)
SiO_3^{2-}	0.34	82
HCO_3^-	2.97	725
SO_4^{2-}	0.50	122
NO_3^-	0.14	34
F^-	0.05	13
$H_2PO_4^-$ / HPO_4^{2-}	0.0013	0.33
Cl^-	1.98	482
Mg^{2+}	0.50	122
Ca^{2+}	0.99	241

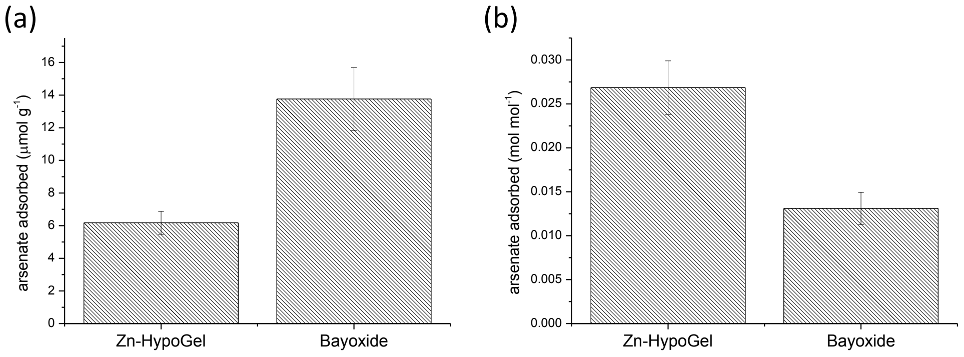

Figure S13 - (a) Number of moles of arsenate adsorbed onto 5 mg of Bayoxide E33 and 5 mg of Zn-HypoGel after 24 hours shaking in 50 ml of Challenge Water at pH 5 and **(b)** number of moles of arsenate adsorbed per mole of active sites available

References

[1] A. E. Hargrove, Z. Zhong, J. L. Sessler, E. V Anslyn, *New J. Chem.* **2010**, *34*, 348–354.

[2] A. L. Spek, *J. Appl. Crystallogr.* **2003**, *36*, 7–13.

[3] H. Adams, D. Bradshaw, D. E. Fenton, *Inorganica Chim. Acta* **2002**, *332*, 195– 200.

[4] H. Adams, D. Bradshaw, D. E. Fenton, *J. Chem. Soc. Dalt. Trans.* **2002**, 925–930.

[5] T.-H. Kwon, H. J. Kim, J.-I. Hong, *Chem. A Eur. J.* **2008**, *14*, 9613–9.
